# Integrated Bulk–Surface Engineering Stabilizes MA‐Free Wide‐Bandgap Perovskites for Tandem Photovoltaics

**DOI:** 10.1002/advs.202520950

**Published:** 2026-01-05

**Authors:** Yu Tong, Biao Li, Youming Zhu, Yehui Wen, Tianchi Zhang, Weihua Ning, Yong Wang, Xuegong Yu, Deren Yang

**Affiliations:** ^1^ Institute of Functional Nano & Soft Materials Joint International Research Laboratory of Carbon‐Based Functional Materials and Devices Soochow University Suzhou P. R. China; ^2^ State Key Laboratory of Silicon and Advanced Semiconductor Materials and School of Materials Science and Engineering Hangzhou Global Scientific and Technological Innovation Center Zhejiang University Hangzhou Zhejiang P. R. China

**Keywords:** bulk–surface molecular coupling effect, defect passivation, halide segregation, tandem photovoltaics, wide‐bandgap perovskite

## Abstract

Optimal wide‐bandgap perovskites are essential for perovskite/silicon tandem solar cells. Conventional wide‐bandgap perovskites, typically FA_1‐_ *_x_*_‐_ *_y_* Cs*_x_* MA*_y_* PbI_1‐_ *_z_* Br*_z_*, contain volatile methylammonium (MA) components and mixed halides that compromise device stability and performance. Removing MA to form FA_1‐_ *_x_* Cs*_x_* PbI_1‐_ *_z_* Br*_z_* eliminates volatile organic components; however, the absence of MA and high Br content required for bandgap widening inevitably accelerates crystallization, increases defect density, and induces severe voltage losses. Here, we present a coupled bulk–surface regulation strategy that fundamentally overcomes these intrinsic bottlenecks. Incorporation of homopiperidinic acid hydroiodide into the precursor heals bulk lattice defects via ─COOH─Pb^2+^ coordination and suppresses halide migration through N─H…I– hydrogen bonding, while subsequent treatment with trimethylenediamine dihydroiodide salts neutralizes surface unsaturated Pb^2+^ and halide vacancies through amino‐Pb^2+^ coordination, hydrogen bonding, and electrostatic interactions. Their coupling effect precisely suppresses defect formation, minimizes non‐radiative recombination, and critically stabilizes halide distribution. As a result, the wide‐bandgap perovskite solar cells achieve an efficiency of 23.71% and enhanced operational stability with T_92_ exceeding 1000 h. Integrated into silicon tandem devices, they deliver 32.26% with long‐term durability. This work establishes molecular coupling consolidation as a new paradigm for constructing stable, high‐efficiency, MA‐free wide‐bandgap perovskites, advancing the practical realization of reliable tandem photovoltaics.

## Graphical abstract

High‐bromide wide‐bandgap perovskites suffer from defects and phase segregation, limiting tandem performance. We report a bulk–surface coupling effect to heal lattice/surface defects and suppress halide migration. As a result, the wide‐bandgap device achieves 23.71% efficiency, while monolithic Si‐based tandems reach 32.26%, exhibiting exceptional long‐term operational stability.

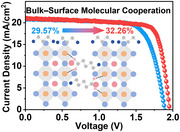

## 1 Introduction

Metal halide perovskites have emerged as one of the most promising next‐generation photovoltaic (PV) materials due to their excellent optoelectronic properties and facile solution processability [1, 2, 3]. Over the past decade, single‐junction perovskite solar cells (PSCs) have rapidly achieved efficiencies exceeding 27% [4, 5, 6]. To surpass the Shockley–Queisser limit, perovskite/silicon tandem solar cells (P/Si TSCs) leverage broad‐spectrum absorption, reaching efficiency approaching 35% [7, 8, 9]. A critical requirement for high‐performance P/Si TSCs is an optimized wide‐bandgap (WBG) perovskite (1.65–1.70 eV). Conventional WBG perovskites, typically FA_1‐_ *_x_*_‐_ *_y_* Cs*_x_* MA*_y_* PbI_1‐_ *_z_* Br*_z_*, contain volatile methylammonium (MA) components and mixed halides, which compromise device stability and performance [10]. Removing MA to form FA_1‐_ *_x_* Cs*_x_* PbI_1‐_ *_z_* Br*_z_* eliminates volatile organic species; however, the absence of MA combined with the high Br content required for bandgap widening accelerates crystallization, increases defect density, and induces severe voltage losses. [11, 12, 13] These intrinsic issues—rapid crystallization, small grains, and abundant grain boundary (GB) defects—exacerbate non‐radiative recombination and phase segregation, ultimately limiting device efficiency and stability.

Stringent control of the internal quality of WBG perovskite is therefore essential. Various strategies —including additive engineering, dimensional regulation, interface modification, etc. — have been developed to suppress halide segregation and passivate defects [14, 15, 16]. For example, precursor additives can tune crystallization kinetics and direct grain growth, mitigating carrier recombination, shunting, and phase segregation [17, 18]. Additives containing lone electron pairs (N, S, and O) can coordinate with uncoordinated Pb^2+^, retard crystallization, reduce defect density, and suppress ion migration [19, 20, 21]. Examples include chloromethylamine for improved crystallinity [22], alkyl ammonium chloride to slow intermediate nucleation [23], and pentamidine hydrochloride to control crystal orientation [24]. Carbonyl‐containing organic additives coordinate strongly with Pb^2+^, reducing deep‐level traps and enhancing PSC performance and stability [25, 26]. Trifluoroacetylamide, for example, leverages amino and halide ions to prevent oxidation while its C═O bonds passivate Pb^2+^ defects. Despite these advances, conventional additives often fail to simultaneously address both bulk and surface/GB defects.

Here, we present a bulk‐surface coupling strategy that simultaneously regulates lattice defects and surface/GB states to realize stable WBG perovskites for P/Si TSCs. In addition to modifying precursor chemistry and regulating crystallization kinetics, comprehensive results indicate that homopiperidinic acid hydroiodide (HAHI) binds uncoordinated Pb^2+^ through ─COOH groups, effectively passivating deep‐level traps and suppressing non‐radiative recombination, while its ─NH_2_ groups form N─H···halide hydrogen bonds that mitigate halide migration. Complementarily, trimethylenediamine dihydroiodide (THDI) passivates Pb^2+^ and I^–^ vacancies as well as surface dangling bonds, through amino–Pb^2+^ coordination, I^–^ ion filling, hydrogen bonding, and electrostatic interactions. Their coupling effect far surpasses their individual contributions: HAHI repairs bulk lattice defects to provide an ideal substrate for THDI surface passivation and band structure optimization, which in turn suppresses bulk ion migration. This dual‐molecule coupling approach precisely modulates crystallization and film morphology, enhances charge transport, reduces non‐radiative recombination, mitigates open‐circuit voltage (*V*_oc_) losses, and suppresses phase segregation in WBG perovskites. Consequently, the optimized 1.68 eV WBG PSCs deliver an efficiency of 23.71% and, more importantly, maintain 92% of their initial efficiency under continuous illumination for over 1000 h. When integrating them into monolithic P/Si TSCs, these devices reach a PCE of 32.26% with a *V*_oc_ of 1.96 V, and a T_92_ lifetime exceeding 900 h. This work establishes molecular synergistic consolidation as a powerful and innovative approach to simultaneously enhance the efficiency and long‐term stability of WBG perovskite‐based tandem photovoltaics.

## 2 Results and Discussion

MA‐free WBG perovskite Cs_0.2_FA_0.8_Pb(I_0.75_Br_0.25_)_3_ with a bandgap of 1.68 eV, was chosen as the research system (denoted as CsFAIBr) to validate our bulk‐surface coupling strategy. HAHI was introduced into the perovskite precursor to regulate growth kinetics, followed by THDI surface mediation. Herein, CsFAIBr with HAHI integration is labeled as B‐CsFAIBr, CsFAIBr with surface THDI mediation is denoted as S‐CsFAIBr, and CsFAIBr with both HAHI integration and surface THDI mediation is designated as BS‐CsFAIBr. Both bulk or/and surface modification had negligible effects on the optical absorption, which maintained absorption edges at ∼738 nm (Figure S1). X‐ray diffractometer (XRD) analysis revealed strong (110) diffraction peaks for all films (Figure S2). Through the analysis of in situ PL data shown in Figure S3, the crystal growth rate of B‐CsFAIBr is significantly slower than that of CsFAIBr. This suggests that HAHI can effectively regulate the crystallization rate during the perovskite crystallization process, which is consistent with the subsequent characterizations. Scanning electron microscopy (SEM) images (Figure S4) show that the average grain size of B‐CsFAIBr increases from 243 to 290 nm, indicating slower growth of perovskite after the integration of HAHI. Subsequent THDI surface‐mediation further enlarges the average grain size to 304 nm (Figure S5), likely due to secondary crystal growth. Atomic force microscopy (AFM) confirms that the BS‐CsFAIBr film exhibits the lowest surface roughness (Figure S6), facilitating improved contact with charge transport layers.

**FIGURE 1:**
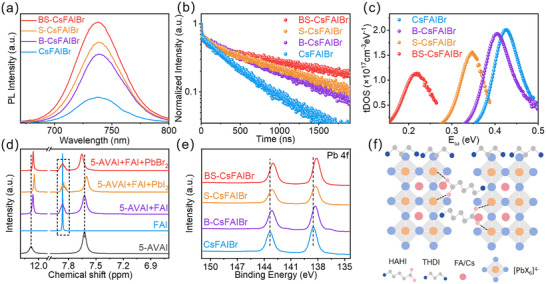
Chemical interaction between HAHI, THDI, and WBG perovskites. (a) Steady‐state PL spectra of perovskite films with different treatments. (b) TRPL spectra of perovskite films with different treatments. (c) Trap density of states (tDOS) results for CsFAIBr, B‐CsFAIBr, S‐CsFAIBr, and BS‐CsFAIBr films. (d) Comparison of ^1^H NMR spectra of HAHI, FAI, HAHI + FAI, HAHI + FAI + PbBr_2_, HAHI + FAI + PbI_2_. (e) XPS spectra under different treatment conditions. (f) Schematic diagram of integrated bulk‐surface engineering.

Comprehensive defect‐related studies indicate that HAHI primarily heals bulk defects, whereas THDI significantly reduces surface defects. Together, they can markedly minimize defects throughout the whole film. As shown in Figure 1a, the steady‐state photoluminescence (PL) intensity increases upon the addition of HAHI integration or THDI surface‐mediation, with the strongest PL observed in BS‐CsFAIBr perovskite films, indicating a reduced defect density. Time‐resolved PL (TRPL) measurements further support this conclusion: the lifetime of carriers for CsFAIBr, B‐CsFAIBr, S‐CsFAIBr, and BS‐CsFAIBr perovskite films are 435.92, 734.80, 785.79, and 1148.94 ns, respectively (Figure 1b). To elucidate the charge transfer process, TRPL decay was analyzed using the differential lifetime, τ_TRPL_(t)  =  −{dln[*Φ*_TRPL_(*t*)]/d(*t*)}^−1^ (Figure S7). The rapid rise of *τ*_TRPL_ within the first ∼500 ns indicates a faster charge transfer in BS‐CsFAIBr perovskite film, indicating significantly improved thin film quality with suppressed defects/traps and better contact with charge transport layers [27].

The defect physics of these films are further investigated (Figure 1c; Figures S8–S11). Thermal admittance spectroscopy (TAS) is performed to probe the trap density and the energy depth of trap states in perovskites. The energy level of trap states deduced from the TAS measurements shows a trap energy depth of 0.43, 0.40, 0.34, 0.22 eV for the CsFAIBr, B‐CsFAIBr, S‐CsFAIBr, and BS‐CsFAIBr films, respectively. Meanwhile, BS‐CsFAIBr films exhibit the lowest defects/traps density (Figure 1c). To further investigate the spatial distribution of trap states in perovskite films, we performed drive‐level capacitance profiling (DLCP) measurements, as shown in Figure S12. The results indicate that surface treatment with THDI can significantly reduce the density of interfacial trap states, while the trap states in the bulk of perovskites can be passivated by HAHI. Moreover, BS treatment can synergistically passivate both the surface and the bulk trap states, which demonstrates that coupling bulk integration with surface mediation provides a moderate passivation effect.

Next, we investigated the defect reduction mechanism on the bulk integration/surface medication. Nuclear magnetic resonance (NMR) hydrogen spectroscopy is first used to investigate the potential chemical interaction between perovskite and these functional molecules. After mixing HAHI with FAI, the resonance signal of protonated ammonium in FAI split from a single peak into two distinct peaks (Figure 1d; Figures S13 and S14), accompanied by chemical shift of the ─NH_3_⁺ and ─COOH protons. By further mixing HAHI + FAI with PbI_2_/PbBr_2_, respectively, two negatively charged oxygen atoms can undergo strong electrostatic interactions or partially coordinated bonds (Pb─O bonds) with Pb^2+^ at the surface or GBs. Fourier transform infrared testing (FTIR) has also demonstrated the role of HAHI in the bulk phase of perovskite, consistent with NMR (Figure S15). These results indicate that I^−^ forms hydrogen bonds with the NH_3_⁺ protons of HAHI, while ─COOH coordinates with Pb^2+^ ions, collectively regulating crystallization and growth (consistent with SEM results) while simultaneously reducing interfacial defects.

Furthermore, the chemical interaction between THDI and perovskites was investigated by NMR hydrogen spectroscopy. Spectra of THDI, FAI, THDI + FAI, THDI + FAI + PbI_2_, THDI + FAI + PbBr_2_ are recorded (Figure S16). Upon the addition of THDI, the amino peaks of FAI exhibit splitting, indicating a change in the chemical environment of the N─H protons. Meanwhile, the amino peaks of THDI also shift, suggesting the formation of N─H···I–hydrogen bonds between THDI and FAI. These interactions enable THDI to form interfacial passivation on the perovskite surface, effectively suppressing ion migration through hydrogen bonding, while simultaneously inducing a potential interfacial dipole layer that optimizes energy‐level alignment and facilitates efficient charge extraction.

To further confirm the chemical interaction between these functional organic molecules and the perovskite crystal, X‐ray photoelectron spectroscopy (XPS) tests are performed on the resulting perovskite films (Figure 1e; Figure S17). The core level of Pb^2+^ and I^–^ peaks of perovskite films shift toward a low binding energy after the solely bulk integration or surface mediation, indicating the strong chemical coupling effect of functional molecule and Pb^2+^/I– in perovskites. Moreover, the bulk‐surface synergistic effect generates a more significant peak shift of the core level of Pb^2+^ and I^–^ peaks, indicating improved binding of Pb^2+^ and I^–^ in perovskites. These changes in binding energy confirm the coupling effect of functional molecules with perovskites, consistent with FTIR and NMR results, ultimately improving the quality of WBG perovskite films.

By integrating the structural features of bimolecular additives and their multiple functional group interactions with perovskites, we elucidate their distinct chemical roles (Figure 1f). Incorporation of HAHI enables its ─NH_2_ and ─COOH groups to coordinate with FA^+^ and free Pb^2+^ ions, regulate crystallization and growth process, simultaneously passivating surface/interface defects, and establishing a hydrogen‐bonding network within the bulk. This process reinforces the crystal lattice, regulates crystal growth, and improves film quality. In parallel, THDI, with its dual ─NH_2_ groups, chemically bonds to surface defect sites, thereby effectively passivating surface defects and suppressing non‐radiative recombination.

**FIGURE 2:**
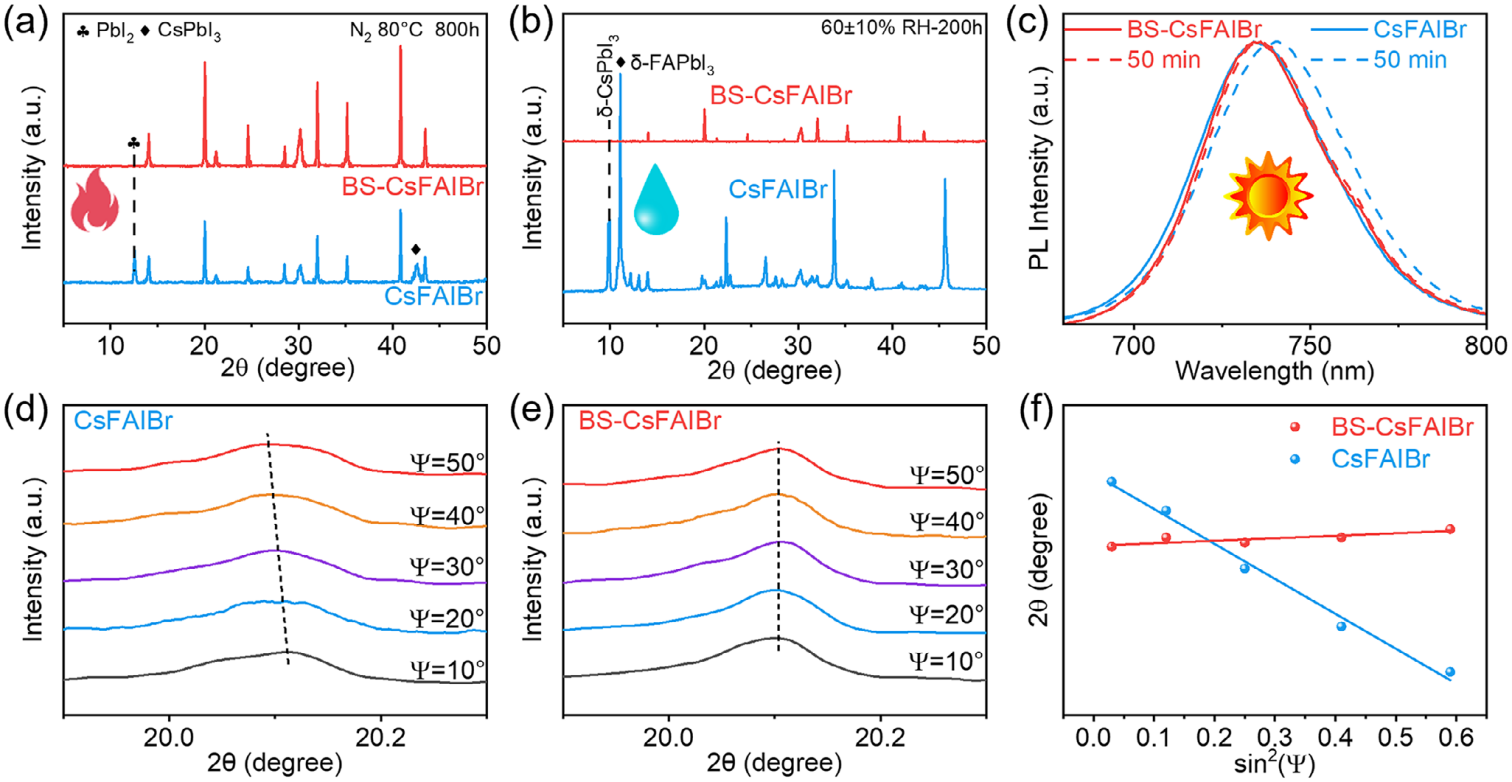
Bulk‐surface molecular coupling methods enhance stability of WBG perovskite films. (a) XRD results of perovskite films heated at 80°C for 800 h in an N_2_ environment. (b) XRD results of perovskite films after 200 h in a 60 ± 10% RH environment. (c) Normalized PL spectra of perovskite films after 50‐min irradiation under 3‐sun intensity. GIXRD spectra at different angles (from 10° to 50°) of (d) CsFAIBr, (e) BS‐CsFAIBr films. (f) Linear fit of the perovskite film obtained by GIXRD is 2θ‐sin^2^(Ψ).

In addition to effectively reducing bulk/surface defects, the bulk‐surface molecular coupling effect can significantly enhance the stability of WBG perovskites. We first evaluated the thermal and moisture stability of the perovskite films. After heating at 80°C in a nitrogen environment for 800 h, BS‐CsFAIBr perovskite film exhibits negligible changes, whereas the CsFAIBr, B‐CsFAIBr, and S‐CsFAIBr films all decomposed with pronounced PbI_2_ formation (Figure 2a; Figure S18). Similarly, when exposed to 60 ± 10% relative humidity (RH) for 200 h, BS‐CsFAIBr preserves its perovskite phase, in contrast to the other films, which show pronounced yellow phases of *δ*‐FAPbI_3_ and *δ*‐CsPbI_3_ (Figure 2b; Figure S19). Collectively, these results demonstrate that BS‐CsFAIBr perovskite films exhibit significantly improved stability under both heating and humidity conditions.

To further elucidate their influence on halide phase segregation, we conducted in situ PL measurements to track phase evolution under continuous light illumination. As shown in Figure 2c, CsFAIBr films exhibit a gradual redshift in PL peak position within 50 min, indicating light‐induced phase segregation. In contrast, BS‐CsFAIBr films maintain nearly invariant PL emission upon continuous light illumination (Figure S20). While those with bulk integration or only surface mediation show moderate shifts (Figure S21). These results confirm that the bulk‐surface molecular coupling effect effectively suppresses light‐induced phase segregation, which is beneficial to improving the operational stability of the devices.

Previous studies have shown that defect reduction is closely linked to enhanced perovskite stability [28, 29, 30]. To further elucidate the origin of the improved stability, we investigated the effect of the bulk‐surface coupling effect on residual strain in perovskite films using grazing‐incidence X‐ray diffraction (GIXRD). As shown in Figure 2d,e and Figure S22, the (110) diffraction peak of the pristine CsFAIBr films shifts toward a smaller angle as the instrument angle (Ψ) increases from 10° to 50°, indicating the presence of lattice strain. In contrast, BS‐CsFAIBr film exhibits negligible peak shifts, suggesting strain relaxation. This trend is further confirmed in the sin^2^ (Ψ) – 2θ plots (Figure 2f; Figure S23), where CsFAIBr film displays a negative slope characteristic of tensile strain, whereas the tensile strain is largely mitigated in the BS‐CsFAIBr films. Such relaxation originates from the dual molecular interactions: ─COOH coordination and hydrogen‐bond networks of HAHI that reinforce the lattice, combined with THDI‐mediated amino coordination at the surface. As a result, the combined reduction of defects, lattice strain relaxation, and hydrogen‐bonding interactions effectively suppresses halide redistribution and light‐induced phase segregation.

Subsequently, the surface potential of the resulting perovskite films was investigated using Kelvin probe force microscopy (KPFM). As shown in Figure S24, both bulk integration and surface mediation increase the contact potential, corresponding to a reduced work function. When combined, the bulk‐surface strategy further lowers the work function, consistent with ultraviolet photoelectron spectroscopy (UPS) results (Figures S25 and S26). Figure S27 shows that BS‐CsFAIBr films achieve optimized band alignment, which minimizes energy loss during charge transfer, promotes efficient charge separation and transport, and thereby enhances device performance [31, 32]. As shown in Figure S28, BS‐CsFAIBr not only has higher device efficiency but also exhibits smaller hysteresis. Overall, the synergistic energy‐level optimization, together with substantial defect suppression, not only improves carrier dynamics but also reduces interfacial recombination, collectively boosting the efficiency and stability of PSCs.

**FIGURE 3:**
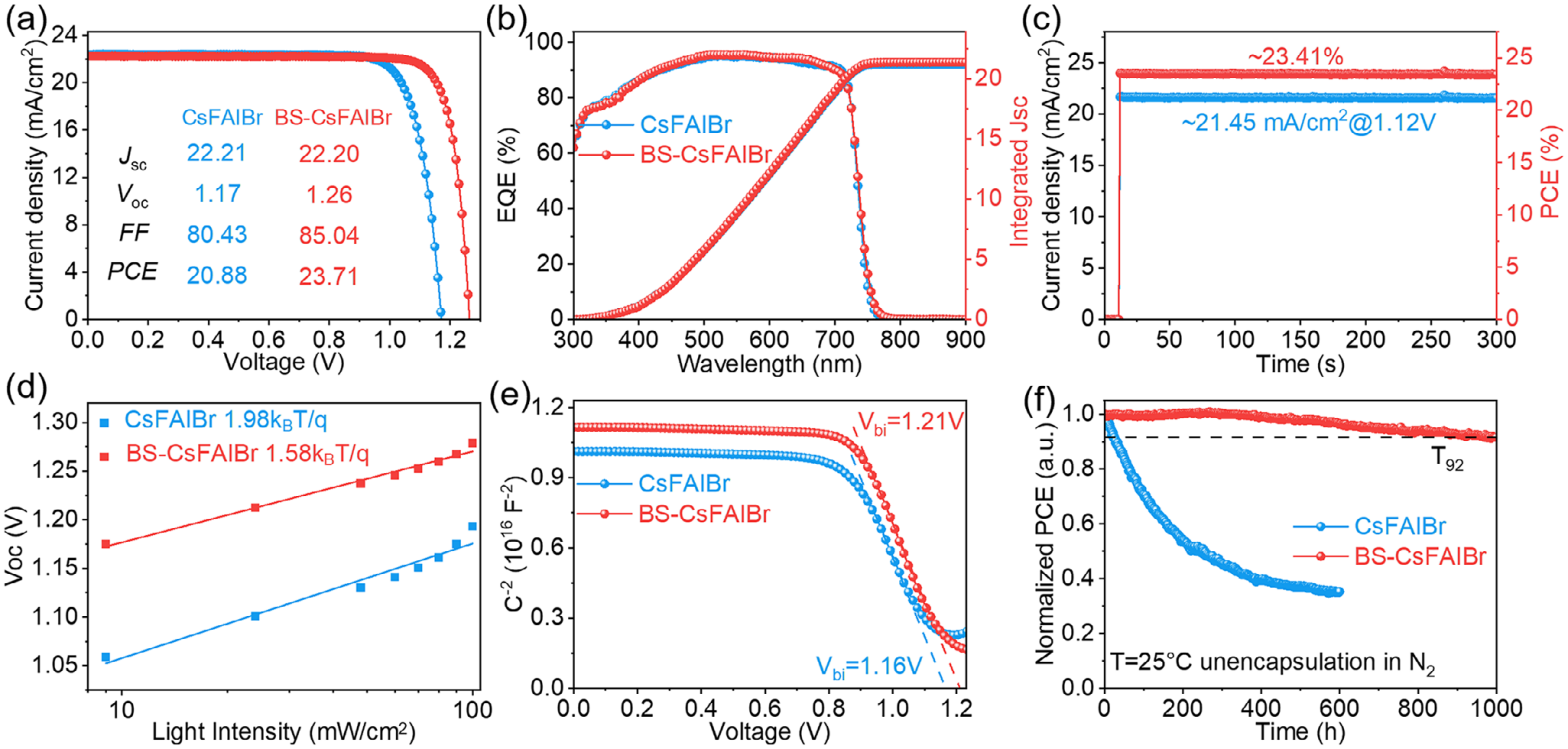
PV performance of PSCs. (a) Typical *J*–*V* curves for CsFAIBr‐ and BS‐CsFAIBr‐ based devices. (b) Corresponding EQE spectra and the integrated *J*_sc_. (c) SPO of CsFAIBr‐ and BS‐CsFAIBr‐ based devices at maximum power point (MPP) tracking. (d) Dependence of *V*_oc_ on light intensity of CsFAIBr‐ and BS‐CsFAIBr‐ based devices. (e) Mott–Schottky plots. (f) Long‐term operational stability of the CsFAIBr‐ and BS‐CsFAIBr device under MPP under continuous 1‐Sun exposure at 25°C N_2_ atmosphere.

We then investigated the effect of bulk‐surface coupling strategy on the device performance of PSCs with a configuration of ITO/SAM/Perovskite/C_60_/BCP/Cu. The champion device based on CsFAIBr films delivers an efficiency of 20.88%, with a short‐circuit current density (*J*_sc_) of 22.21 mA cm^−2^, an open‐circuit voltage (*V*_oc_) of 1.17 V, and a fill factor (FF) of 80.43%. Either B‐CsFAIBr or S‐CsFAIBr‐based devices demonstrate improved device parameters, while BS‐CsFAIBr‐based device achieves a significantly enhanced PCE of 23.71%, with a *J*_SC_ 22.20 mA cm^−2^, a *V*_OC_ of 1.26 V, and *FF* of 85.04% (Figure 3a; Figure S29). Statistical analysis of the PV parameters further confirms substantial improvement in performance for the bulk‐surface molecular coupling strategy (Figure S30). The radiative recombination limit *FF* (*FF_SQ_*) calculated according to the Shockley–Queisser model, the calculated pseudo *FF* (*pFF*, *FF* without carrier transport losses), and the measured *FF* of the device (*FF* measured) are presented in Figure S31. The results indicate that, compared to optimizing only the bulk (B) or the surface (S), the performance improvement is not comprehensive. However, for devices treated on both B and S, the performance losses are significantly reduced. Given that the bulk‐surface molecular coupling strategy results in the most pronounced enhancement, subsequent investigations primarily focus on CsFAIBr and BS‐CsFAIBr‐based devices. The external quantum efficiency (EQE) spectra of CsFAIBr and BS‐CsFAIBr devices exhibit integrated *J*_sc_ values consistent with those obtained from the *J*–*V* curves (Figure 3b). Figure 3c shows the stable power output (SPO) of the BS‐CsFAIBr‐based device at the maximum power point, with a stable PCE of 23.41% at a bias voltage of 1.12 V.

Bulk‐surface coupling strategy significantly improves *V*_oc_, primarily by suppressing non‐radiative recombination, in line with previous photophysical measurements on the films that revealed significantly reduced defects/traps density in the films. Further measurements on the devices also reach similar conclusions. To evaluate the suppression of non‐radiative recombination, we examined the device response under different light intensities. Both CsFAIBr and BS‐CsFAIBr devices exhibit a linear dependence (Figure 3d); however, the ideality factors were 1.98 and 1.58 for CsFAIBr and BS‐CsFAIBr‐based devices, indicating a substantial reduction of defect‐assisted recombination with the synergistic strategy [33, 34]. Mott–Schottky [35, 36] analysis further revealed built‐in potentials of 1.21 V in the BS‐CsFAIBr device compared to 1.16 V for CsFAIBr (Figure 3e), suggesting more efficient charge separation. Defect density was further quantified using space‐charge‐limited current (SCLC) measurements. For electron‐only devices (ITO/ SnO_2_/ Perovskite/ C_60_/ BCP/ Cu), the trap‐filled limit voltage decreased from 0.305 V in the CsFAIBr to 0.141 V in the BS‐CsFAIBr (Figure S32a), confirming a significantly reduced defect density. A similar trend was observed for the hole‐only device (Figure S32b). Dark current testing further demonstrated that the BS‐CsFAIBr device exhibited nearly two orders of magnitude lower dark current compared with CsFAIBr (Figure S33), consistent with reduced defect density, lower leakage current, and suppressed non‐radiative recombination, ultimately benefiting *V*_oc_ improvement [37].

Device stability was also assessed under maximum power point tracking (MPPT) using 1‐sun illumination at 25°C. The BS‐CsFAIBr device maintains 92% of its initial PCE (T_90_) after over 1000 h of continuous operation, whereas the CsFAIBr device reached T_90_ after only 100 h (Figure 3f). In summary, improved film quality, enhanced charge separation and transfer, and suppressed non‐radiative recombination collectively contribute to the remarkable enhancement in operational stability of the dual‐passivated devices.

**FIGURE 4:**
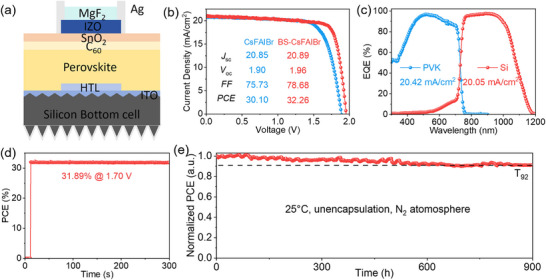
Optoelectronic performance of silicon/perovskite stacked tandem cells. (a) Schematic diagram of our P/Si TSCs. (b) *J*–*V* curve of champion P/Si TSC based on optimized WBG perovskite. (c) EQE spectra of the corresponding champion P/Si TSCs. (d) The stable output power (SPO) of the champion P/Si TSCs. (e) SPO tracking stability for champion P/Si TSCs.

We first fabricated a transparent single‐junction (S‐J) PSC using the device stack of indium tin oxide (ITO)/4PADCB/Perovskite/modification layer/C_60_/SnO_2_/indium‐doped zinc oxide (IZO)/ silver (Ag). The thicknesses of C_60_, SnO_2_, and IZO were 20, 17, and 40 nm, respectively. Under reverse *J*–*V* scans, the semi‐transparent structure showed a PCE of 21.59% (*V*_OC_ of 1.25 V, *J*_SC_ of 20.87 mA cm^−2^, *FF* of 82.50%) with no noticeable hysteresis (Figure S34). The results indicate that, compared to conventional devices, the semi‐transparent PSCs did not exhibit significant open‐circuit voltage (*V*_OC_) loss. This suggests that our sputtering process did not introduce additional losses, which is beneficial for fabricating high‐efficiency tandem devices. To further verify the effectiveness of optimized WBG perovskite application in P/Si TSCs, we used silicon heterojunction solar cells as the bottom cells with textured front and back to fabricate tandem devices. The schematic diagram of the silicon/perovskite tandem structure is shown in Figure 4a. CsFAIBr‐based TSC delivers an efficiency of 30.10%, with a *J*_sc_ of 20.85 mA cm^−2^, a *V*_oc_ of 1.90 V, and a *FF* of 75.73%, while BS‐CsFAIBr‐based devices demonstrate a significantly improved efficiency of 32.26%, with a *V*_oc_ of 1.96 V, a *J*_sc_ of 20.89 mA cm^−2^, and an *FF* of 78.68% (Figure 4b). The *J*_sc_ values obtained by the EQE integration of the top WBG subcell and the bottom silicon subcell are 20.42 and 20.05 mA cm^−2^, respectively (Figure 4c), which are in good agreement with the *J*_sc_ values measured by *J*–*V* tests. The representative device shows a stabilized PCE of 31.89% as shown in the SPO result (Figure 4d). In addition, the P/Si TSC exhibits good long‐term stability, maintaining 92% of the initial PCE after more than 900 h tests (Figure 4e). These remarkable findings illustrate the effectiveness of the bulk‐surface molecular coupling strategy in WBG perovskites, contributing to the realization of efficient and stable P/Si TSCs.

## 3 Conclusion

In all, we demonstrate a bulk–surface coupling effect—precursor‐incorporated HAHI and surface‐mediated by THDI—that concurrently heals bulk lattice defects (─COOH─Pb^2+^ coordination, N─H···halide hydrogen bonds) and passivates surface traps (amino–Pb^2+^ coordination, iodide filling, electrostatic interactions). Their cooperative action precisely controls crystallization, suppresses non‐radiative recombination and halide migration, and prevents light‐induced phase segregation. Consequently, single‐junction WBG PSCs deliver an efficiency of 23.71% with a *V*_oc_ of 1.26 V, while integration into monolithic Si/perovskite tandems achieves over 32% efficiency with a *V*_oc_ of 1.96 V. This bulk–surface coupling strategy establishes a powerful route for overcoming intrinsic limitations of WBG perovskites, underscoring the critical role of precise defect and crystallization control in advancing next‐generation perovskite and tandem photovoltaics.

## 4 Experimental Section

### 4.1 Materials

Formamidiniumiodide (FAI), Cesium iodide (CsI), lead (II) iodide (PbI_2_), Lead bromide (PbBr_2_), (4‐(7H‐dibenzo[c, g]carbazol‐7‐yl)butyl)phosphonic Acid (4PADCB), homopiperidinic acid hydroiodide, trimethylenediamine dihydroiodide, N,N‐dimethylformamide (DMF, 99.8%), dimethyl sulfoxide (DMSO, 99.7%), chlorobenzene (CB, 99.8%), and isopropanol (IPA 99.8% anhydrous) were purchased from Sigma–Aldrich., C60 and BCP (>99% sublimed) were purchased from Xi'an Polymer Light Technology Corp.

### 4.2 Perovskite Precursor Solution

Perovskite precursor solution (1.4 m) was prepared by dissolving 192.6 mg FAI, 72.8 mg CsI, 192.7 mg PbBr_2_, 403.5 mg PbI_2_ in 1 mL mixed DMF and DMSO solvents (volume ratio: 4:1) and stirred in a N_2_ glovebox at room temperature overnight before film fabrication.

### 4.3 Preparation of Thin Film and Perovskite Solar Cells

ITO glass was sequentially ultrasonicated with water, ethanol, and IPA, and treated with UV ozone for 20 min before use. For the various perovskite films used for testing, the following deposition method was used to deposit the perovskite film directly on the ITO substrate, and the direction of the incident light from the surface of the perovskite film. To fabricate solar cells, a 4PACDB IPA solution at a concentration of 0.3 mg mL^−1^ was spin‐coated onto ITO glass at a rotational speed of 5000 rpm. After cooling, 80 µL of perovskite precursor was spin‐coated with an acceleration of 1000 rpm and rotation for 35 s at a speed of 5000 rpm. Among them, HAHI at a concentration of 1 mg mL^−1^ was added to the perovskite precursor solution. Drop 200 µL of CB onto the membrane 7 s before closing. Samples were annealed on a hot plate at 100°C for 30 min, and then 100 µL of THDI solution containing 1 mg mL^−1^ was first coated on a perovskite membrane with rotation at 5000 rpm for 30 s, then annealed at 100°C for 10 min. All steps were performed at room temperature in an N_2_ glove box. Finally, C60 (40 nm), BCP (7 nm), and Cu (100 nm) were sequentially deposited on the perovskite surface by thermal evaporation. At a temperature of 100°C and a lamination time of 10 min, the PSCs were encapsulated using a laminator. Polyolefin elastomer (POE) film was used as the encapsulation material, and the edges were sealed with butyl rubber to ensure a strong and moisture‐resistant barrier.

### 4.4 Thin Film Characterization

Top view obtained by scanning electron microscopy (ZEISS Gemini SEM 500). XRD spectra were obtained with a D8Discover (Bruker, Germany) diffractometer under Cu K/rfs (<s:2> = 1.54 Å) irradiation. Ultraviolet photoelectron spectroscopy (UPS) and X‐ray photoelectron spectroscopy (XPS) were measured using the PHI5000 VersaProbe III with an X‐ray spot size of 500 µm. The UV–Vis spectrum of 400 ∼ 900 nm was collected by a Cary 60 UV–vis spectrophotometer (Agilent, Germany). High‐resolution 1H NMR data were recorded on a Bruker NEO 600 MHz spectrometer with Larmor frequency V(1H) = 600.35 MHz. Dried d6‐DMSO was used as a solvent and was also used to calibrate isotropic chemical shifts. The temperature was maintained at 294 ± 0.2 K at the time of measurement. KPFM images were acquired by Dimension XR scanning probe microscopy (Bruker, USA). Steady‐state PL and TRPL were measured with a FLS1000 photoluminescence spectrometer (Edinburgh Instruments Ltd.) with an excitation laser at 430 nm. TRPL was detected at a wavelength of 736 nm.

### 4.5 Silicon/Perovskite Tandem Device Manufacturing

Wide bandgap perovskite top cells were deposited on a silicon substrate. The ITO composite layer was used as the intermediate composite layer. The preparation process of the perovskite layer was the same as that of the single‐junction device. After thermal evaporation of the C_60_ layer, a layer of 17 nm of tin oxide (SnO_2_, tetrakis(dimethylamino)tin, and H_2_O as the precursors, with N_2_ as the gas carrier) was deposited via ALD (Picosun) with 140 cycles and used as the protective buffer layer. A 40 nm indium zinc oxide (IZO) target was prepared by radio frequency magnetron sputtering at room temperature and deposited in pure argon gas. The 500 nm silver electrode was thermally evaporated through the designed mask at a rate of 2.0 Å s^−1^ as a metal raster, and the silver electrode was deposited on the backside. Finally, ∼130 nm of MgF_2_ was thermally evaporated at a rate of 1.0 Å s^−1^ as the reflection layer.

### 4.6 Device Characteristics

The current density–voltage (*J*–*V*) characteristics of the device were measured with a Keithley 2400 source meter under simulated AM1.5 G solar irradiation at room temperature. Standard silicon cells were used to calibrate the light intensity. The cell area was 0.1 cm^2^, and a mask of 0.087 cm^2^ was used prior to testing. Forward and reverse scans (from −0.1 to 1.3 V) and positive and negative scans (from 1.3 to −0.1 V) to measure the *J*–*V* curve at a scan rate = 50 mV s^−1^. EQE data were obtained by QE‐R (Enlitech). The dark *I*–*V* characteristics of SCLC measurements of ITO/SnO_2_/perovskite/C_60_/BCP/Ag structural devices were recorded using a Keithley 2400 source meter. Operational stability was measured by placing the device in a nitrogen chamber with continuous white LED illumination (equal to 1 solar intensity). Under simulated continuous AM1.5 G illumination (LED, 100 mW cm^−2^), the maximum power point (MPP) tracking was carried out using a solar cell aging test system (Shenzhen Lancheng Technology Co., Ltd.). *J*–*V* characteristics of the tandem devices were measured with a Keithley 2401 source meter under the simulated AM 1.5 G illumination (100 mW cm^−2^) using an Enlitech simulation light source (SS‐PST100D) with variable standard spectral (reserve scan: 2 V—(−0.1 V), forward scan: (−0.1 V)—2 V, scan rate: 0.05 V S^−1^). The light intensity was calibrated by SRC‐2020‐KG3‐RTD, SRC‐2020‐BL7‐RTD, and SRC‐2020‐QTZ‐RTD stand Si cell before the tandem test.

## Conflicts of Interest

The authors declare no conflicts of interest.

## Supporting information




**Supporting File 1**: advs73663‐sup‐0001‐SuppMat.docx.

## Data Availability

The data that support the findings of this study are available from the corresponding author upon reasonable request.

## References

[1] S. De Wolf , J. Holovsky , S. J. Moon , et al., “Organometallic Halide Perovskites: Sharp Optical Absorption Edge and its Relation to Photovoltaic Performance,” The Journal of Physical Chemistry Letters 5 (2014): 1035–1039, 10.1021/jz500279b. 26270984

[2] M. Alexandre , M. Chapa , S. Haque , et al., “Optimum Luminescent Down‐Shifting Properties for High Efficiency and Stable Perovskite Solar Cells,” ACS Applied Energy Materials 2 (2019): 2930–2938, 10.1021/acsaem.9b00271.

[3] N. Li , Z. L. Zhu , J. W. Li , A. K. Y. Jen , and L. D. Wang , “Inorganic CsPb_1−x_Sn_x_IBr_2_ for Efficient Wide‐Bandgap Perovskite Solar Cells,” Advanced Energy Materials 8 (2018): 1800525.

[4] H. Chen , C. Liu , J. Xu , et al., “Improved Charge Extraction in Inverted Perovskite Solar Cells With Dual‐Site‐Binding Ligands,” Science 384 (2024): 189–193, 10.1126/science.adm9474. 38603485

[5] A. Kojima , K. Teshima , Y. Shirai , and T. Miyasaka , “Organometal Halide Perovskites as Visible‐Light Sensitizers for Photovoltaic Cells,” Journal of the American Chemical Society 131 (2009): 6050–6051, 10.1021/ja809598r. 19366264

[6] H. Min , D. Lee , J. Kim , et al., “Perovskite Solar Cells With Atomically Coherent Interlayers on SnO_2_ Electrodes,” Nature 598 (2021): 444–450, 10.1038/s41586-021-03964-8. 34671136

[7] J. P. Mailoa , C. D. Bailie , E. C. Johlin , et al., “A 2‐Terminal Perovskite/Silicon Multijunction Solar Cell Enabled by a Silicon Tunnel Junction,” Applied Physics Letters 106 (2015): 121105.

[8] S. P. Bremner , M. Y. Levy , and C. B. Honsberg , “Analysis of Tandem Solar Cell Efficiencies Under AM1.5G Spectrum Using a Rapid Flux Calculation Method,” Progress in Photovoltaics: Research and Applications 16 (2008): 225–233, 10.1002/pip.799.

[9] Z. S. Yu , M. Leilaeioun , and Z. Holman , “Selecting Tandem Partners for Silicon Solar Cells,” Nat Energy 1 (2016): 16137.

[10] E. Aydin , T. G. Allen , M. De Bastiani , et al., “Interplay Between Temperature and Bandgap Energies on the Outdoor Performance of Perovskite/Silicon Tandem Solar Cells,” Nature Energy 5 (2020): 851–859, 10.1038/s41560-020-00687-4.

[11] E. L. Unger , L. Kegelmann , K. Suchan , D. Sörell , L. Korte , and S. Albrecht , “Correction: Roadmap and Roadblocks for the Band Gap Tunability of Metal Halide Perovskites,” Journal of Materials Chemistry A 5 (2017): 15983–15983, 10.1039/C7TA90141K.

[12] Y. Tong , A. Najar , L. Wang , et al., “Wide‐Bandgap Organic‐Inorganic Lead Halide Perovskite Solar Cells,” Advanced Science 9 (2022): 2105085. 35257511 10.1002/advs.202105085 PMC9109050

[13] Q. Ye , B. J. Fan , Y. L. Zhou , et al., “Competitive Crystallization Modulated Phase‐Homogeneous Wide‐Bandgap Perovskites for Monolithic Perovskite‐Organic Tandem Solar Cells,” Advanced Materials 37 (2025): 11781. 10.1002/adma.202511781 40838425

[14] X. F. Huang , Y. L. Hou , Q. F. Feng , et al., “Spontaneously Healing Buried Interfaces in n–i–p Halide Perovskite Photovoltaics,” Advanced Energy and Sustainability Research 4 (2023): 2200150.

[15] K. Wei , L. Yang , J. D. Deng , Z. D. Luo , and X. L. Zhang , “Facile Exfoliation of the Perovskite Thin Film for Visualizing the Buried Interfaces in Perovskite Solar Cells,” ACS Applied Energy Materials 5 (2022): 7458–7465, 10.1021/acsaem.2c00948.

[16] C. Luo , G. H. J. Zheng , F. Gao , et al., “Engineering the Buried Interface in Perovskite Solar Cells via Lattice‐Matched Electron Transport Layer,” Nature Photonics 17 (2023): 856–864, 10.1038/s41566-023-01247-4.

[17] Q. Jiang , J. H. Tong , R. A. Scheidt , et al., “Compositional Texture Engineering for Highly Stable Wide‐Bandgap Perovskite Solar Cells,” Science 378 (2022): 1295–1300, 10.1126/science.adf0194. 36548423

[18] M. C. Brennan , S. Draguta , P. V. Kamat , and M. Kuno , “Light‐Induced Anion Phase Segregation in Mixed Halide Perovskites,” ACS Energy Letters 3 (2018): 204–213, 10.1021/acsenergylett.7b01151.

[19] X. Z. Wang , D. C. Liu , R. C. Liu , et al., “PbI_6_ Octahedra Stabilization Strategy Based on π‐π Stacking Small Molecule Toward Highly Efficient and Stable Perovskite Solar Cells,” Advanced Energy Materials 13 (2023): 2203635.

[20] K. W. Yeom , D. K. Lee , and N. G. Park , “Hard and Soft Acid and Base (HSAB) Engineering for Efficient and Stable Sn‐Pb Perovskite Solar Cells,” Advanced Energy Materials 12 (2022): 2202496.

[21] Q. F. Feng , X. F. Huang , Z. H. Tang , et al., “Governing PbI_6_ Octahedral Frameworks for High‐Stability Perovskite Solar Modules,” Energy & Environmental Science 15 (2022): 4404–4413, 10.1039/D2EE02162E.

[22] M. Kim , G. H. Kim , T. K. Lee , et al., “Methylammonium Chloride Induces Intermediate Phase Stabilization for Efficient Perovskite Solar Cells,” Joule 3 (2019): 2179–2192, 10.1016/j.joule.2019.06.014.

[23] J. Park , J. Kim , H. S. Yun , et al., “Controlled Growth of Perovskite Layers With Volatile Alkylammonium Chlorides,” Nature 616 (2023): 724–730, 10.1038/s41586-023-05825-y. 36796426

[24] P. J. Shi , Y. Ding , B. Ding , et al., “Oriented Nucleation in Formamidinium Perovskite for Photovoltaics,” Nature 620 (2023): 323–327, 10.1038/s41586-023-06208-z. 37344595

[25] S. Y. Wang , S. X. Wang , J. R. Wang , et al., “Achieving 20% Efficiency in Organic Solar Cells Through Conformationally Locked Solid Additives,” Advanced Energy Materials (2025): 2405205, 10.1002/aenm.202405205.

[26] S. Zhan , Y. W. Duan , Z. K. Liu , et al., “Stable 24.29%‐Efficiency FA_0.85_MA_0.15_PbI_3_ Perovskite Solar Cells Enabled by Methyl Haloacetate‐Lead Dimer Complex,” Advanced Energy Materials 12 (2022): 2200867.

[27] C. X. Kan , P. J. Hang , S. B. Wang , et al., “Efficient and stable perovskite‐silicon tandem solar cells with copper thiocyanate‐embedded perovskite on textured silicon,” Nature Photonics 19 (2025): 63–70, 10.1038/s41566-024-01561-5.

[28] H. Zai , P. Yang , J. Su , et al., “Wafer‐Scale Monolayer MoS_2_ Film Integration for Stable, Efficient Perovskite Solar Cells,” Science 387 (2025): 186–192, 10.1126/science.ado2351. 39787220

[29] Y. Zhang , Q. Z. Song , G. L. Liu , et al., “Improved Fatigue Behaviour of Perovskite Solar Cells With an Interfacial Starch–Polyiodide Buffer Layer,” Nature Photonics 17 (2023): 1066–1073, 10.1038/s41566-023-01287-w.

[30] W. X. Zhang , X. M. Guo , Z. B. Cui , et al., “Strategies for Improving Efficiency and Stability of Inverted Perovskite Solar Cells,” Advanced Materials 36 (2024): 2311025, 10.1002/adma.202311025. 38427593

[31] S. Q. Fu , G. Li , S. Zhou , et al., “Synergistic Bimolecular Erosion‐Healing Interfacial Passivation for Wide‐Bandgap Perovskite and Tandem Solar Cells,” Science Bulletin 70 (2025): 1786–1792, 10.1016/j.scib.2025.04.017. 40253291

[32] G. L. Wang , W. Y. Duan , Q. Lian , et al., “Reducing Voltage Loss via Dipole Tuning for Electron‐Transport in Efficient and Stable Perovskite‐Silicon Tandem Solar Cells,” Advanced Energy Materials 14 (2024): 2401029.

[33] S. Solak , P. W. M. Blom , and G. A. H. Wetzelaer , “Effect of Non‐Ohmic Contacts on the Light‐Intensity Dependence of the Open‐Circuit Voltage in Organic Solar Cells,” Applied Physics Letters 109 (2016): 053302.

[34] P. Caprioglio , C. M. Wolff , O. J. Sandberg , et al., “On the Origin of the Ideality Factor in Perovskite Solar Cells,” Advanced Energy Materials 10 (2020): 2000502.

[35] E. H. Jung , N. J. Jeon , E. Y. Park , et al., “Efficient, Stable and Scalable Perovskite Solar Cells Using Poly(3‐Hexylthiophene),” Nature 567 (2019): 511–515, 10.1038/s41586-019-1036-3. 30918371

[36] K. Gelderman , L. Lee , and S. W. Donne , “Flat‐Band Potential of a Semiconductor: Using the Mott–Schottky Equation,” Journal of Chemical Education 84 (2007): 685, 10.1021/ed084p685.

[37] C. W. Li , Z. N. Song , D. W. Zhao , et al., “Reducing Saturation‐Current Density to Realize High‐Efficiency Low‐Bandgap Mixed Tin–Lead Halide Perovskite Solar Cells,” Advanced Energy Materials 9 (2019): 1803135.

