# Supplementary material for: Integrated Bulk–Surface Engineering Stabilizes MA‐Free Wide‐Bandgap Perovskites for Tandem Photovoltaics
Source: Adv Sci (Weinh). 2026 Jan 5;13(16):e20950. doi: 10.1002/advs.202520950 (PMC13042856; doi:10.1002/advs.202520950)
Supplement: Supplementary file 1 — Supporting File 1: advs73663‐sup‐0001‐SuppMat.docx. [file ADVS-13-e20950-s001.docx]

((Supporting Information can be included here using this template))

Supporting Information

Integrated Bulk–Surface Engineering Stabilizes MA-Free Wide-Bandgap Perovskites for Tandem Photovoltaics

Yu Tong, Biao Li, Youming Zhu, Yehui Wen, Tianchi Zhang, Weihua Ning*, Yong Wang*, Xuegong Yu, Deren Yang


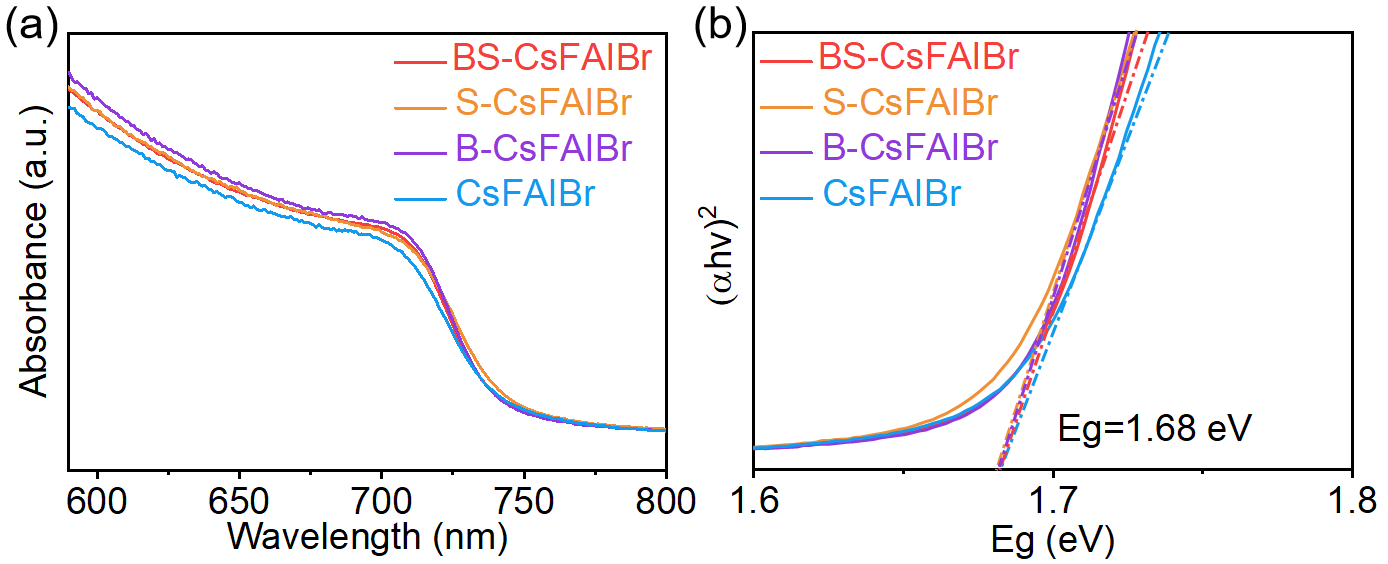


**Figure S1.** a) UV-vis absorption spectra and b) Tauc plots of the perovskite films.

**
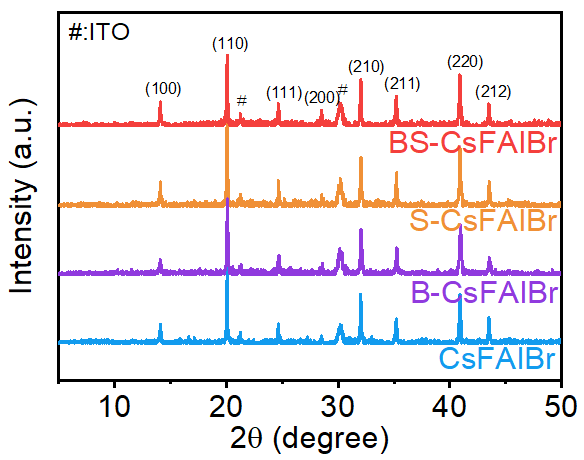
**

**Figure S2.** XRD diffraction patterns of CsFAIBr, B-CsFAIBr, S-CsFAIBr, and BS-CsFAIBr, respectively.


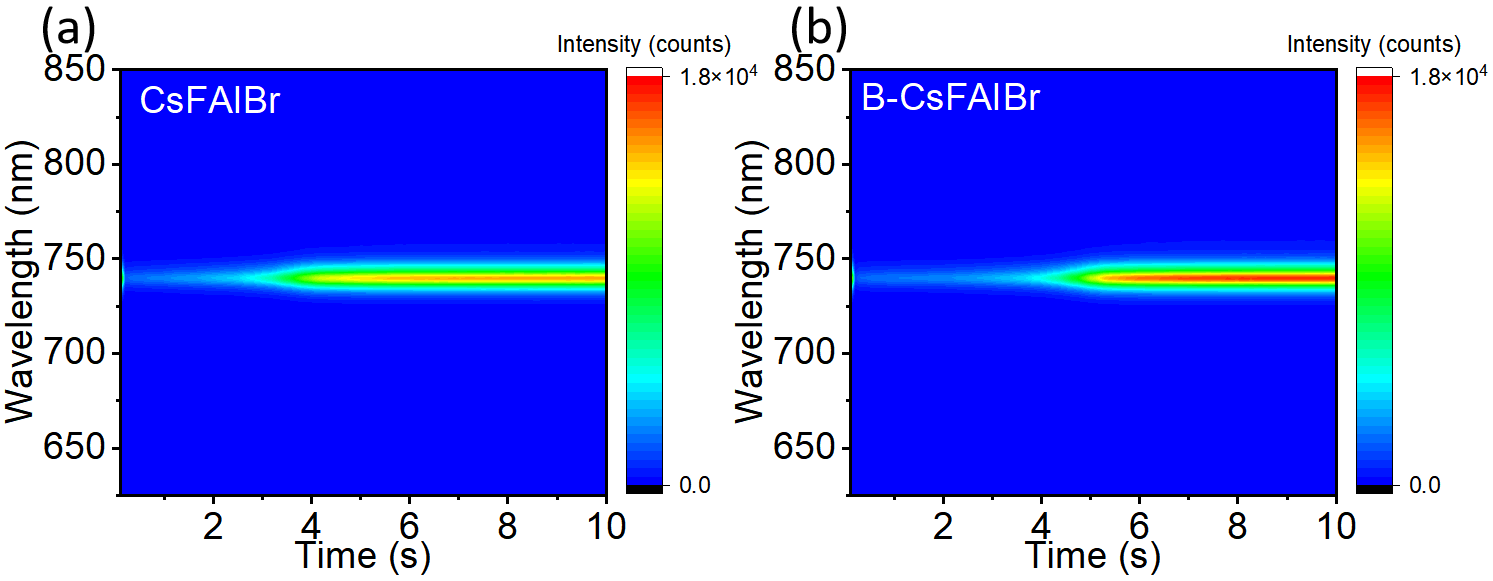


**Figure S3.** In situ PL spectra of the a) CsFAIBr and b) B-CsFAIBr perovskite films.

**
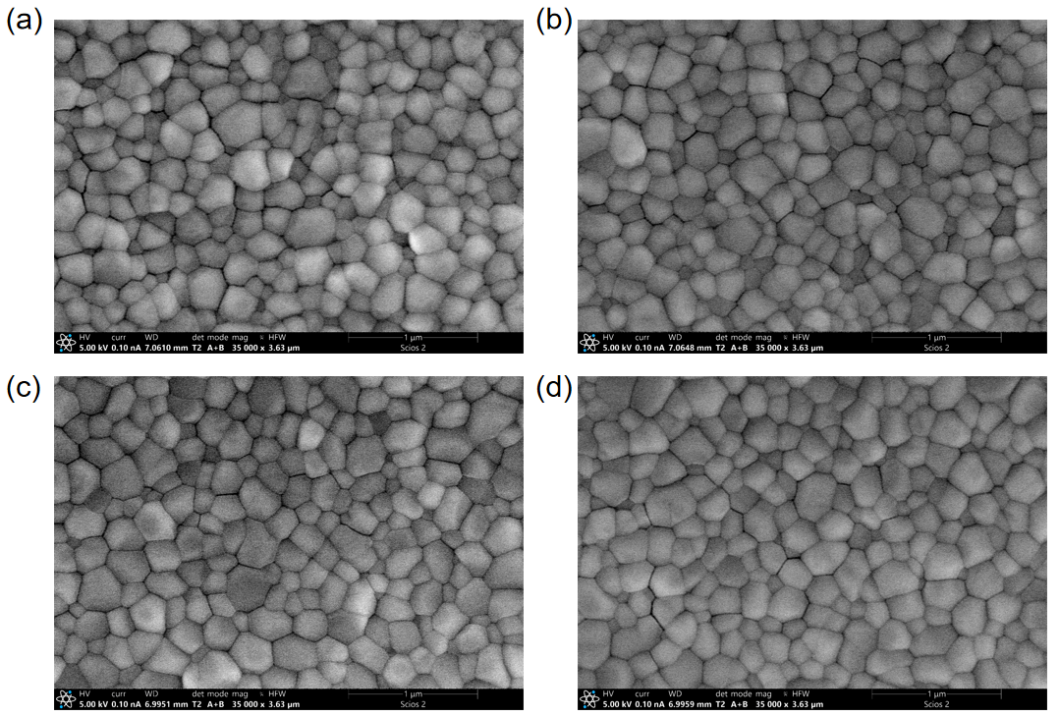
**

**Figure S4.** SEM images of the a) CsFAIBr, b) S-CsFAIBr, c) B-CsFAIBr, d) BS-CsFAIBr perovskite films.

**
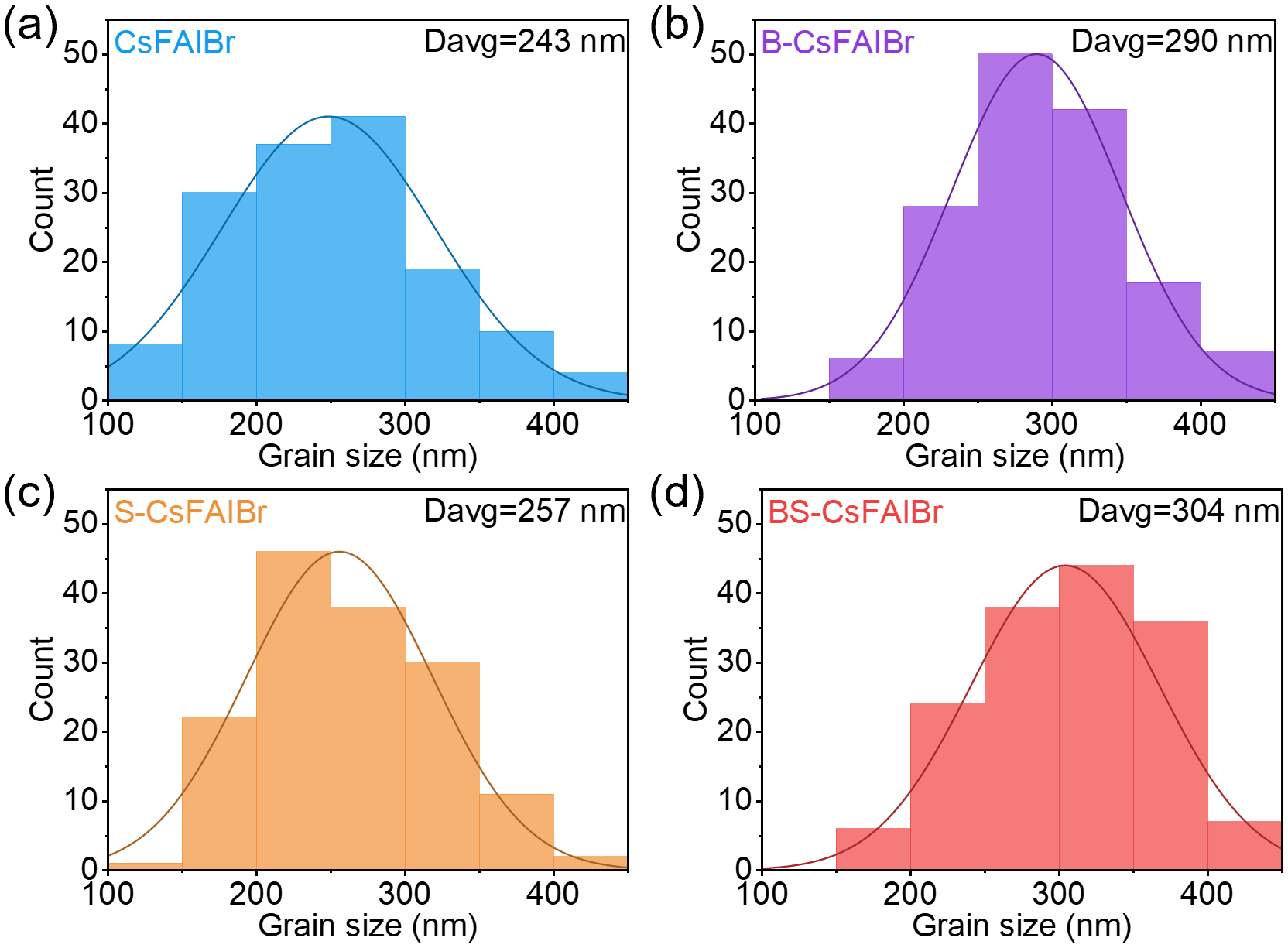
**

**Figure S5.** statistical chart of grain size of the a) CsFAIBr, b) B- CsFAIBr, c) S- CsFAIBr, d) BS- CsFAIBr perovskite films.

**
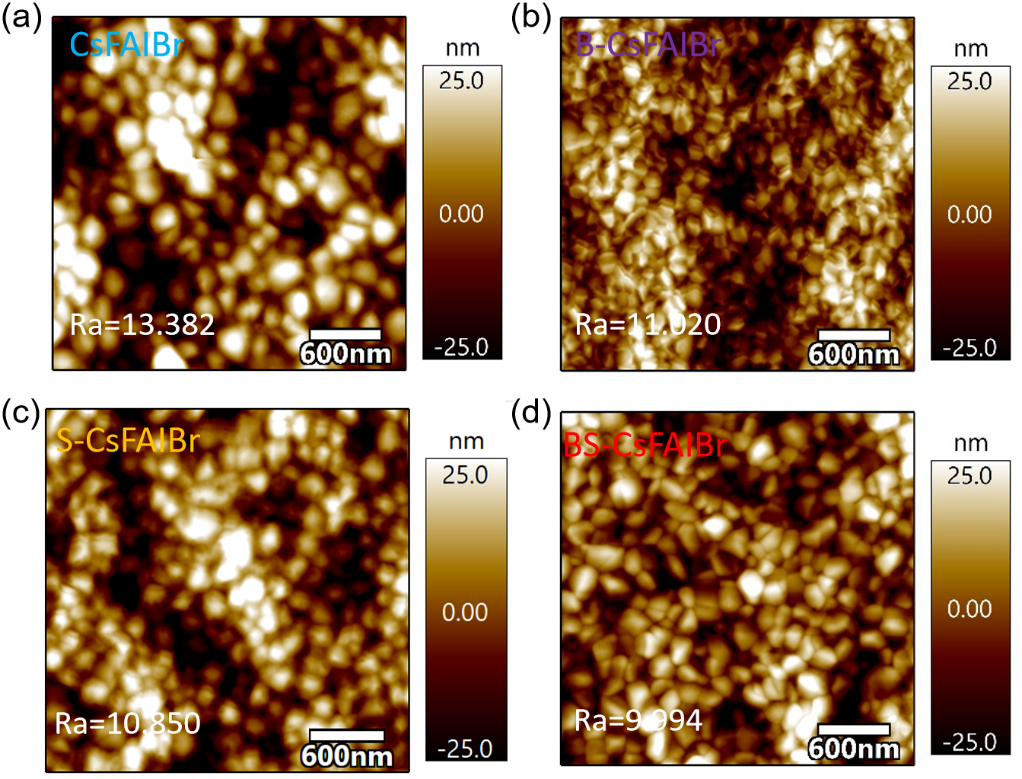
**

**Figure S6.** Atomic force probe microscopy (AFM) images of perovskite films.

**
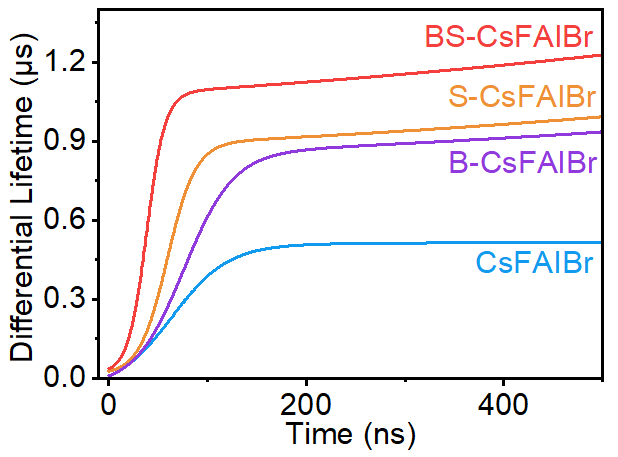
**

**Figure S7.** Differential lifetimes of perovskite films.

**
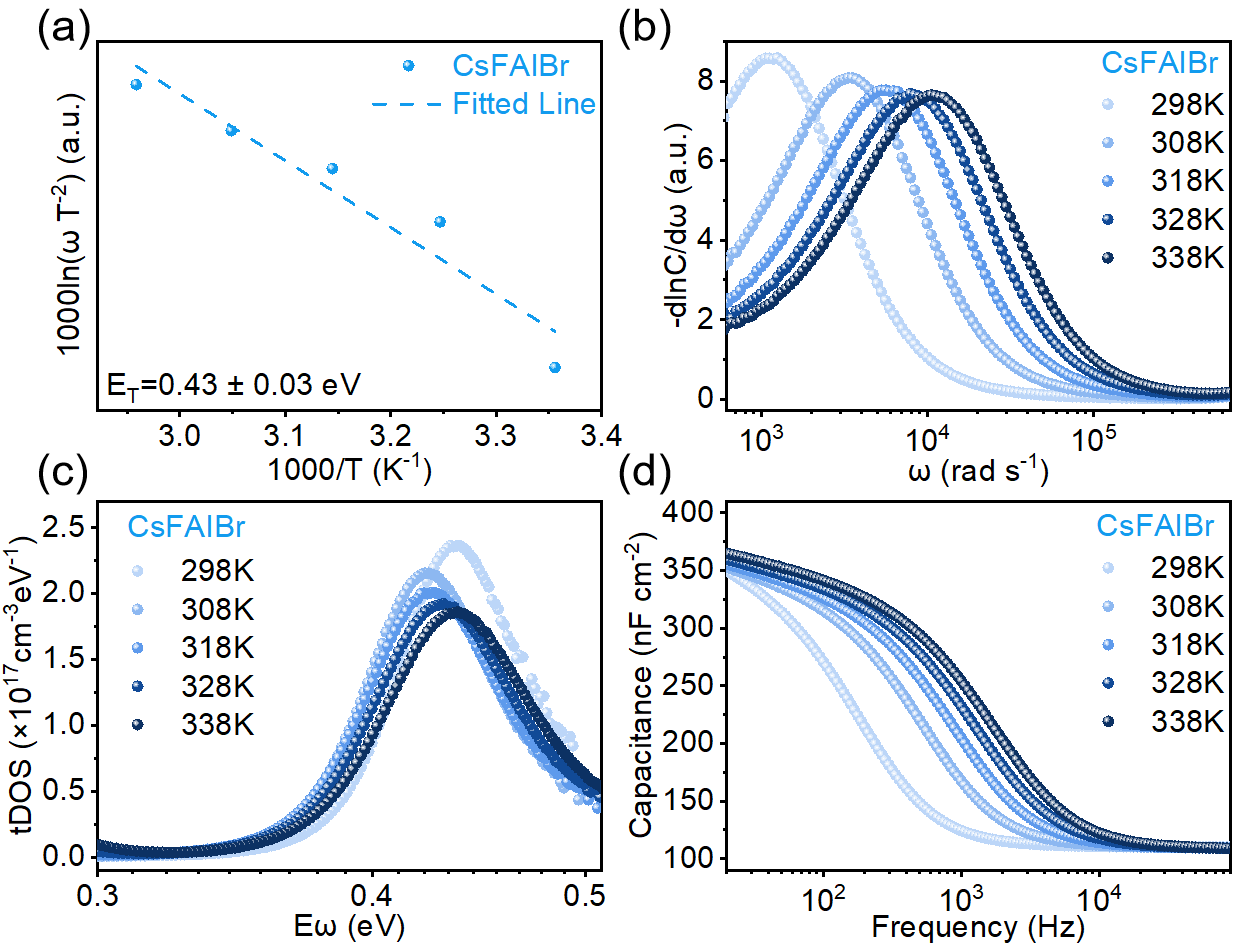
**

**Figure S8.** a) Arrhenius curves of the CsFAIBr films. b) The −dln*C*/d*ω* curve of the films at different temperatures (298 K–338 K) with angular frequency *ω*. c) tDOS of the films at different temperatures. d) Capacitance-frequency (*C*-*f*) curves of the films at different temperatures.

**
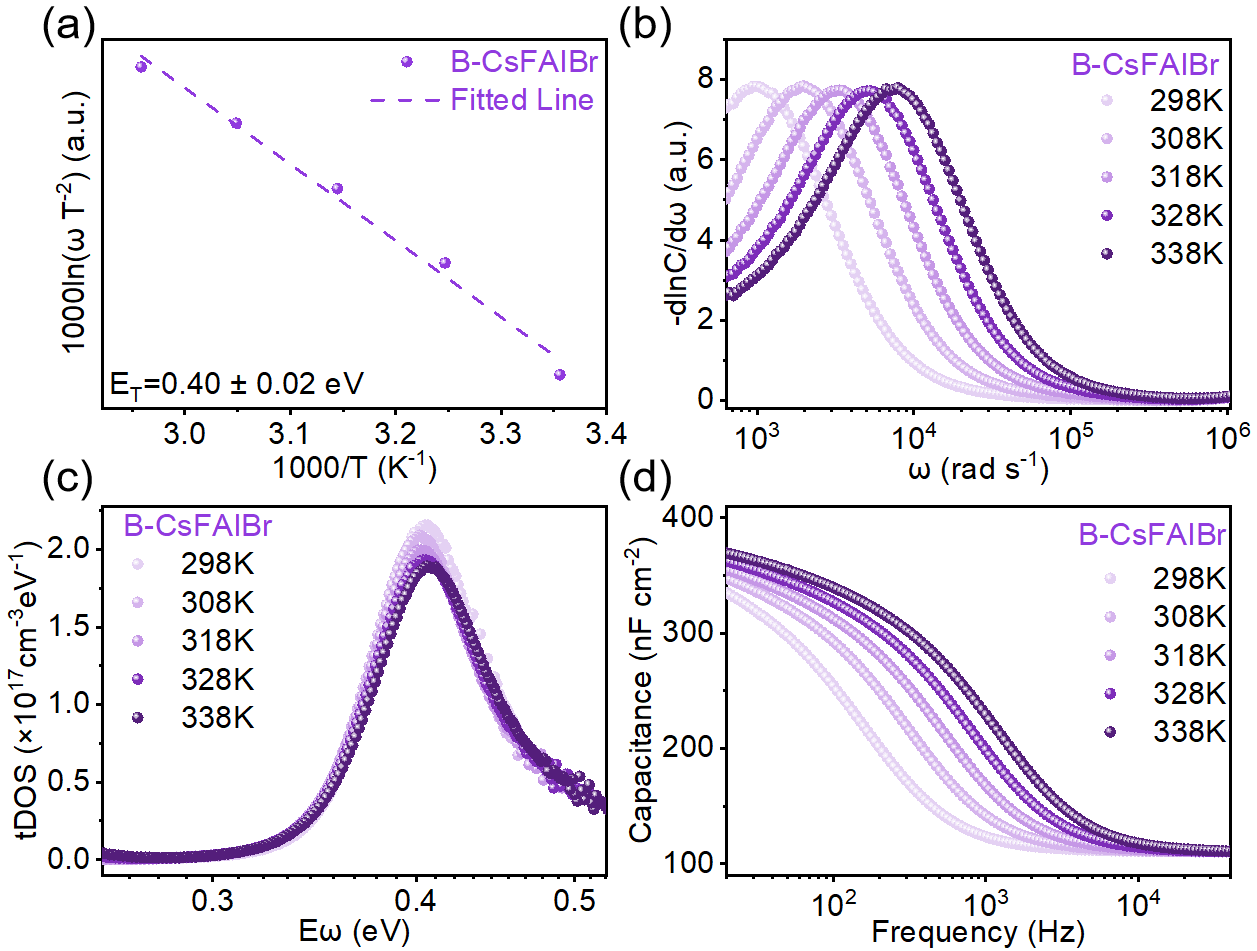
**

**Figure S9.** a) Arrhenius curves of the B-CsFAIBr films. b) The −dln*C*/d*ω* curve of the films at different temperatures (298 K–338 K) with angular frequency *ω*. c) tDOS of the films at different temperatures. d) *C*-*f* curves of the films at different temperatures.

**
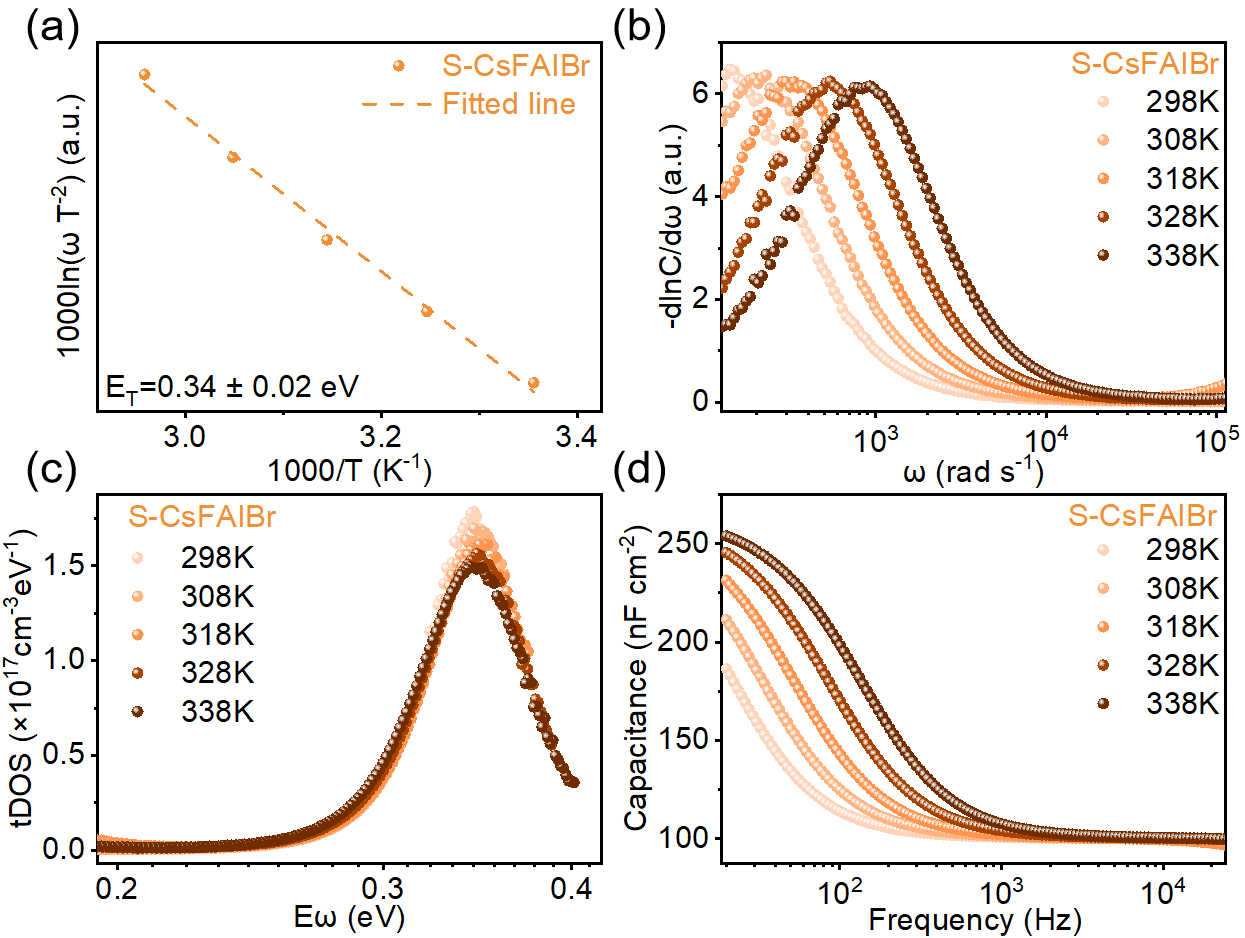
**

**Figure S10.** a) Arrhenius curves of the S-CsFAIBr films. b) The −dln*C*/d*ω* curve of the films at different temperatures (298 K–338 K) with angular frequency *ω*. c) tDOS of the films at different temperatures. d) *C*-*f* curves of the films at different temperatures.

**
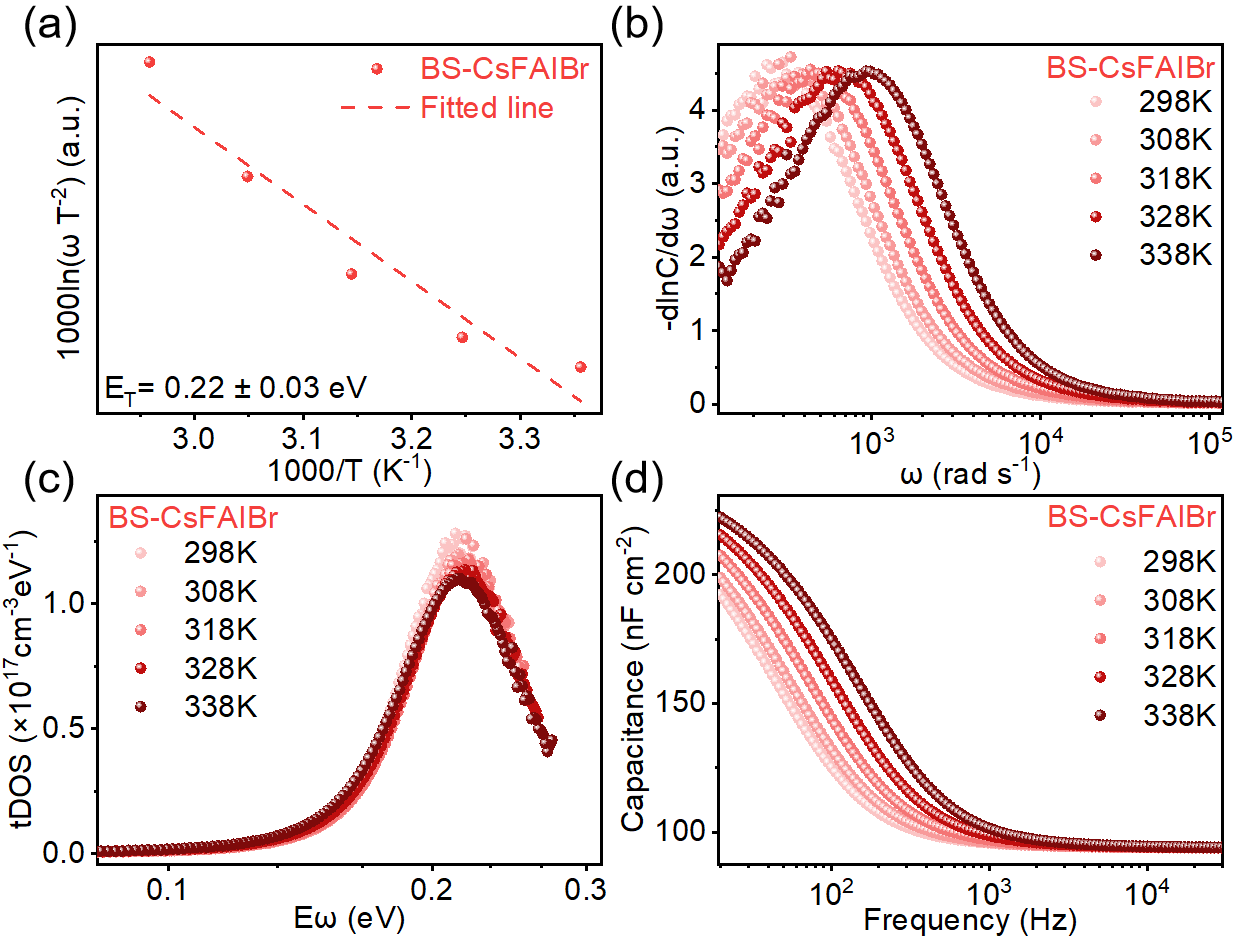
**

**Figure S11.** a) Arrhenius curves of the BS-CsFAIBr films. b) The −dln*C*/d*ω* curve of the films at different temperatures (298 K–338 K) with angular frequency *ω*. c) tDOS of the films at different temperatures. d) *C*-*f* curves of the films at different temperatures.

**
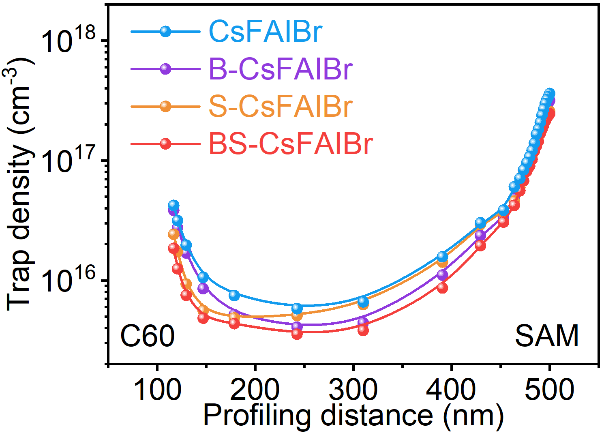
**

**Figure S12.** DLCP Study of Four Devices under Different Processing Conditions (Profiling distances 0 and 500 nm represent the C60/perovskite and perovskite/ 4PADCB interfaces, respectively).

**
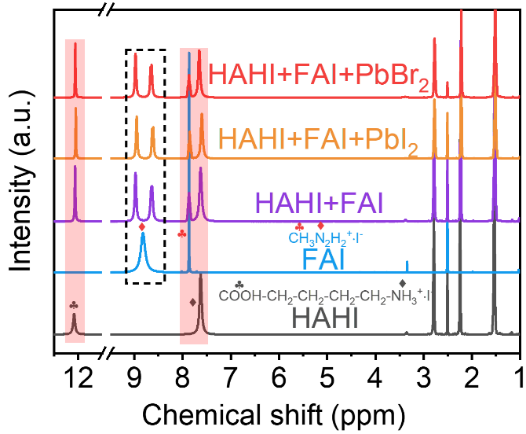
**

**Figure S13.** Comparison of NMR hydrogen spectra of HAHI, FAI, HAHI+FAI, HAHI+FAI+PbBr_2_, HAHI+ FAI+PbI_2_.

**
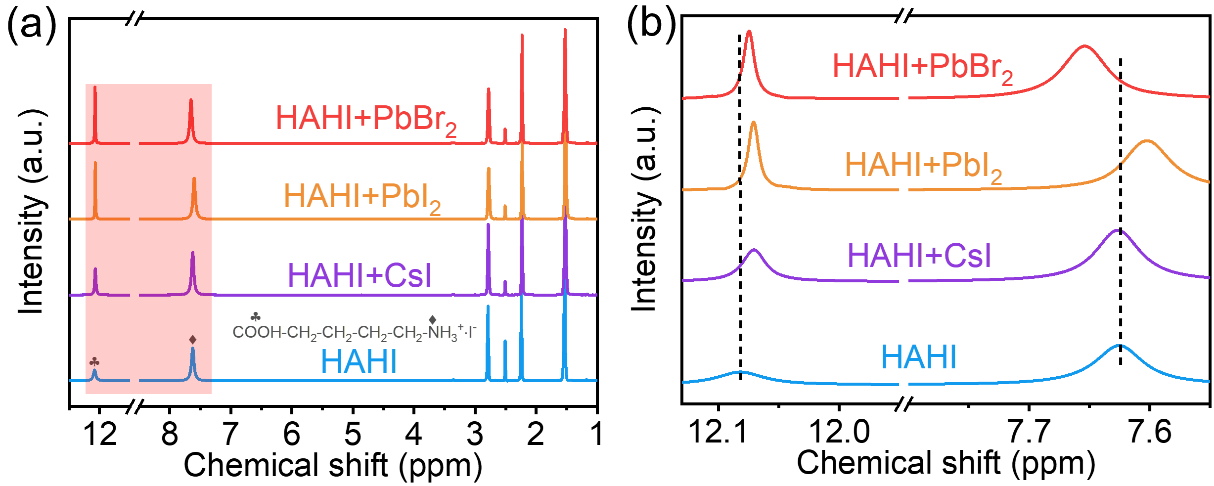
**

**Figure S14.** a) Comparison of NMR hydrogen spectra of HAHI, HAHI + CsI, HAHI + PbBr_2_, HAHI + PbI_2_ with b) enlarged image in the shaded area in (a).

**
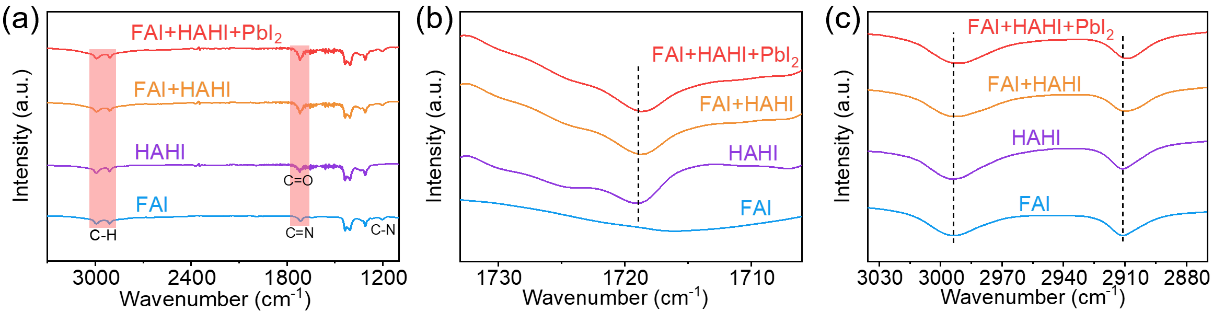
**

**Figure S15.** (a) FTIR spectra of FAI, HAHI, FAI+HAHI and FAI+HAHI+PbI_2_, (b) and (c) enlarged image in the shaded area in (a).

**
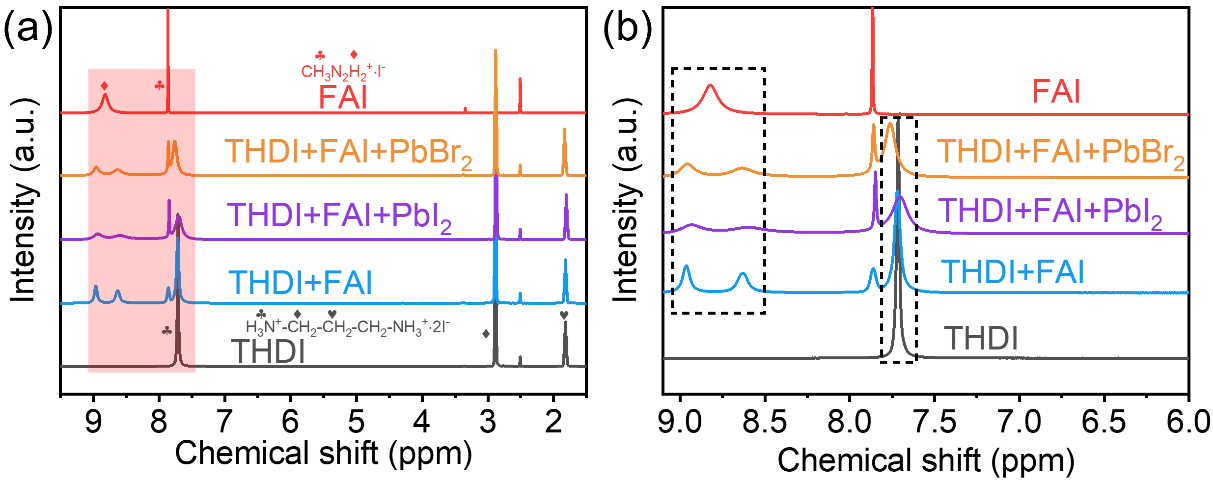
**

**Figure S16.** a) Image of comparison of NMR hydrogen spectra of THDI, FAI, THDI+FAI, THDI+FAI+PbBr_2_, THDI+FAI+PbI_2_ and b) enlarged image in the shaded area in (a).

**
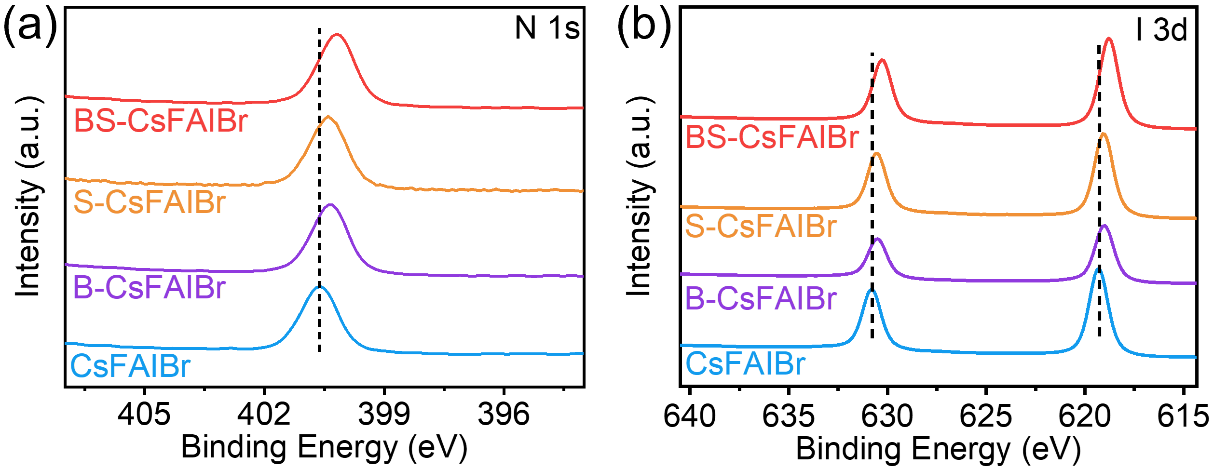
**

**Figure S17.** High-resolution XPS spectra of N 1s and I 3*d* of the CsFAIBr, B-CsFAIBr, S-CsFAIBr, and BS-CsFAIBr perovskite films.

**
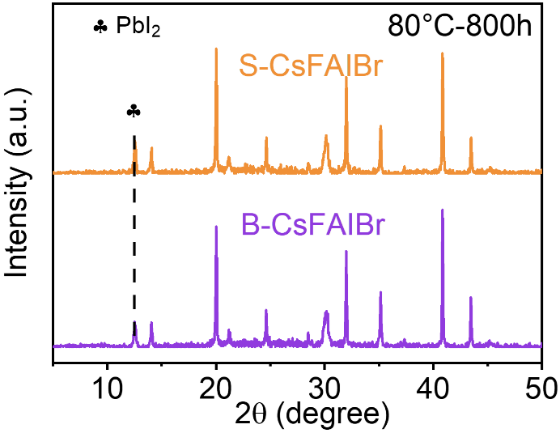
**

**Figure S18.** XRD results of perovskite films heated at 80°C for 800 h in an N_2_ environment.

**
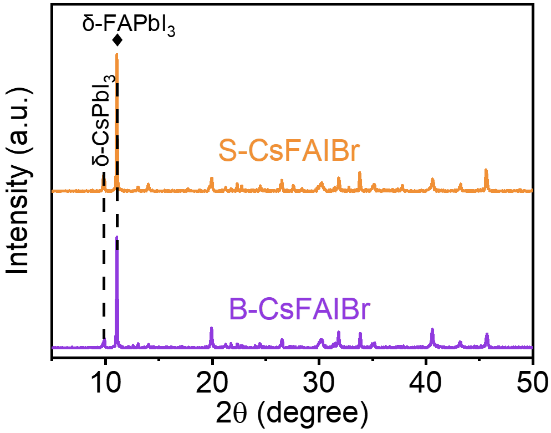
**

**Figure S19.** XRD results of perovskite films after 200 h in a 60 ± 10% RH environment.

**
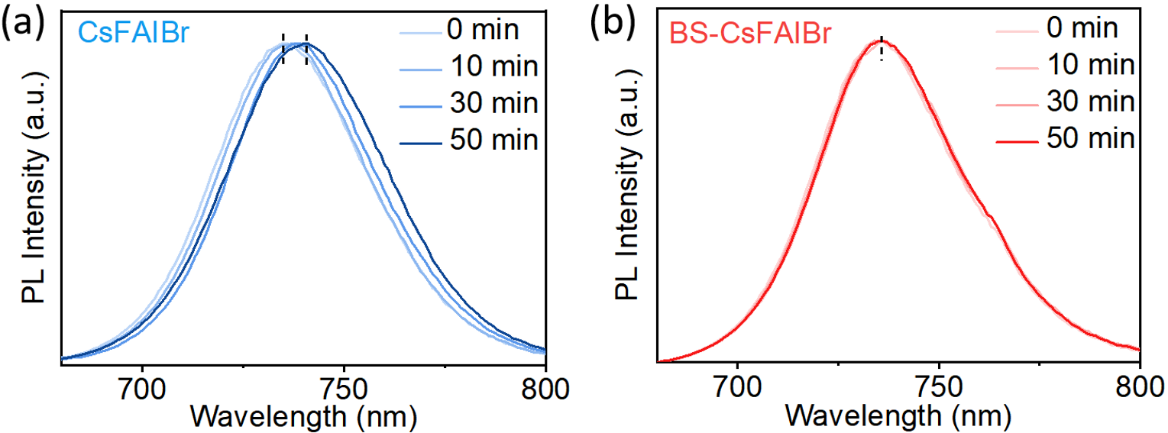
**

**Figure S20.** Normalized photoluminescence (PL) spectra of perovskite films with a) CsFAIBr. b) BS-CsFAIBr before and after 50 min of 3 sun intensity irradiation.

**
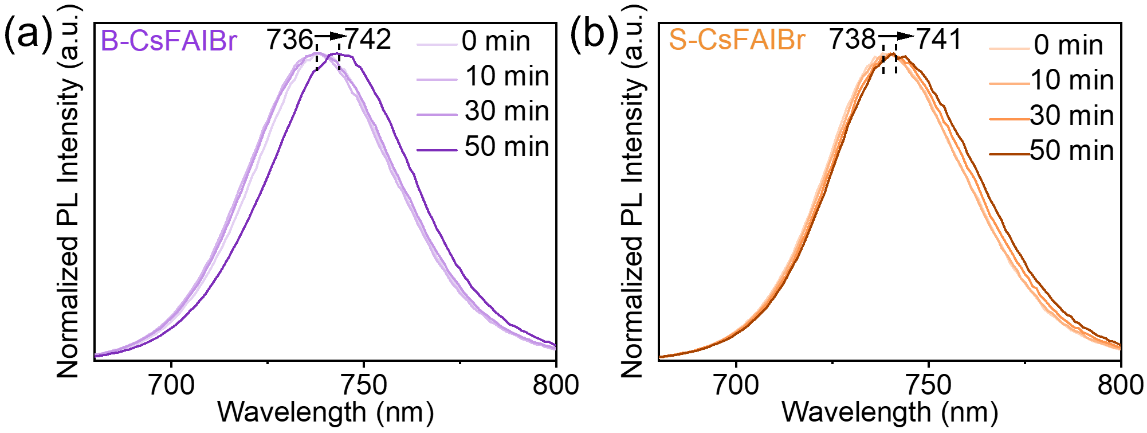
**

**Figure S21.** Normalized photoluminescence (PL) spectra of perovskite films with a) B-CsFAIBr. b) S-CsFAIBr before and after 50 min of 3 sun intensity irradiation.

**
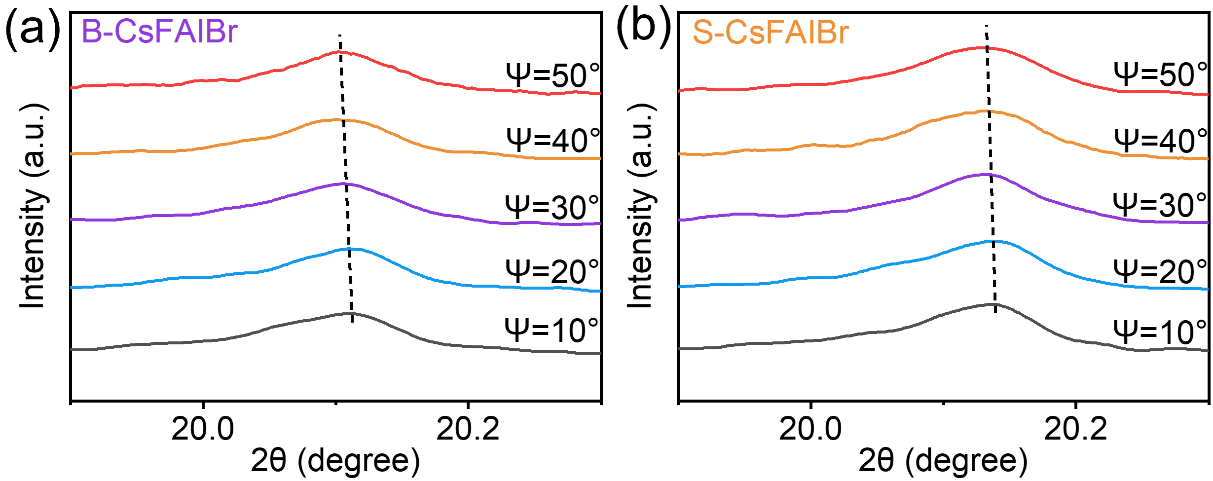
**

**Figure S22.** GIXRD spectra at different angles (from 10° to 50°) of a) B-CsFAIBr, b) S-CsFAIBr films.

**
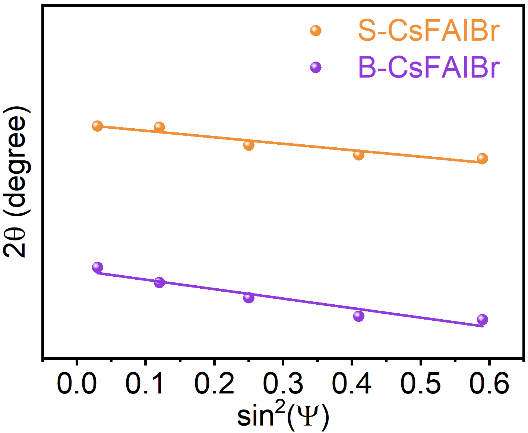
**

**Figure S23.** Linear fit of the perovskite film obtained by GIXRD is 2θ-sin^2^ (Ψ).

**
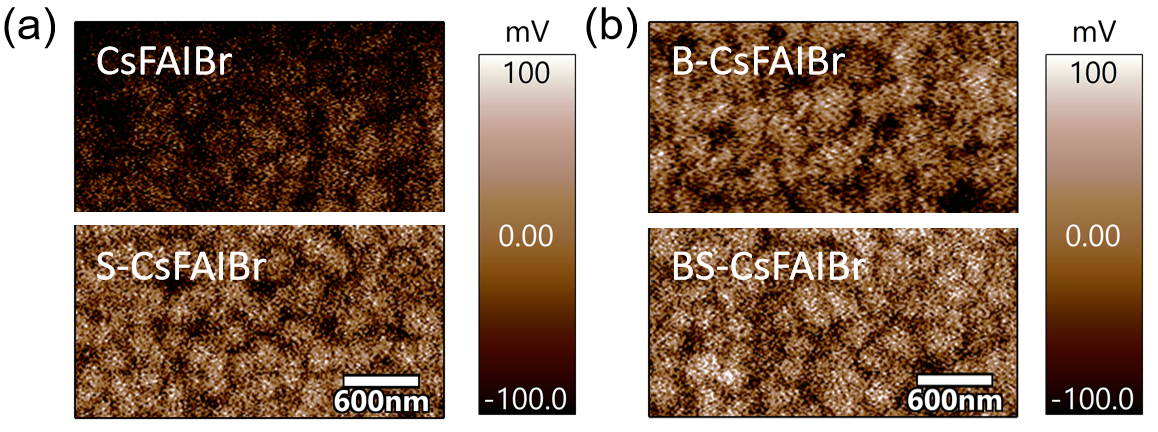
**

**Figure S24.** a, b) Kelvin probe force microscopy (KPFM) images of perovskite films.

**
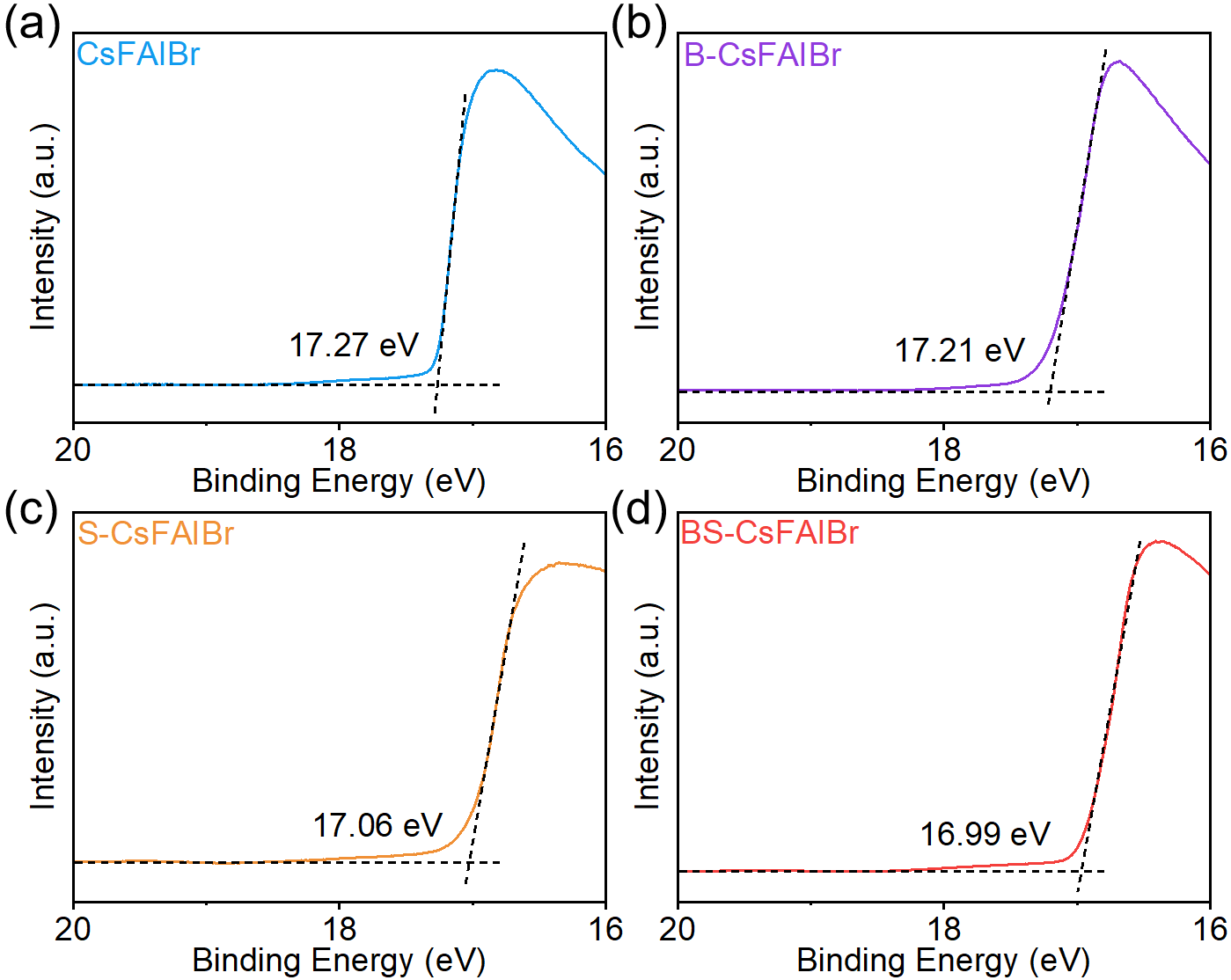
**

**Figure S25.** UPS spectra (using the He-I line with photon energy of 21.22 eV) of the as-prepared CsFAIBr, B-CsFAIBr, S-CsFAIBr, and BS-CsFAIBr films.

**
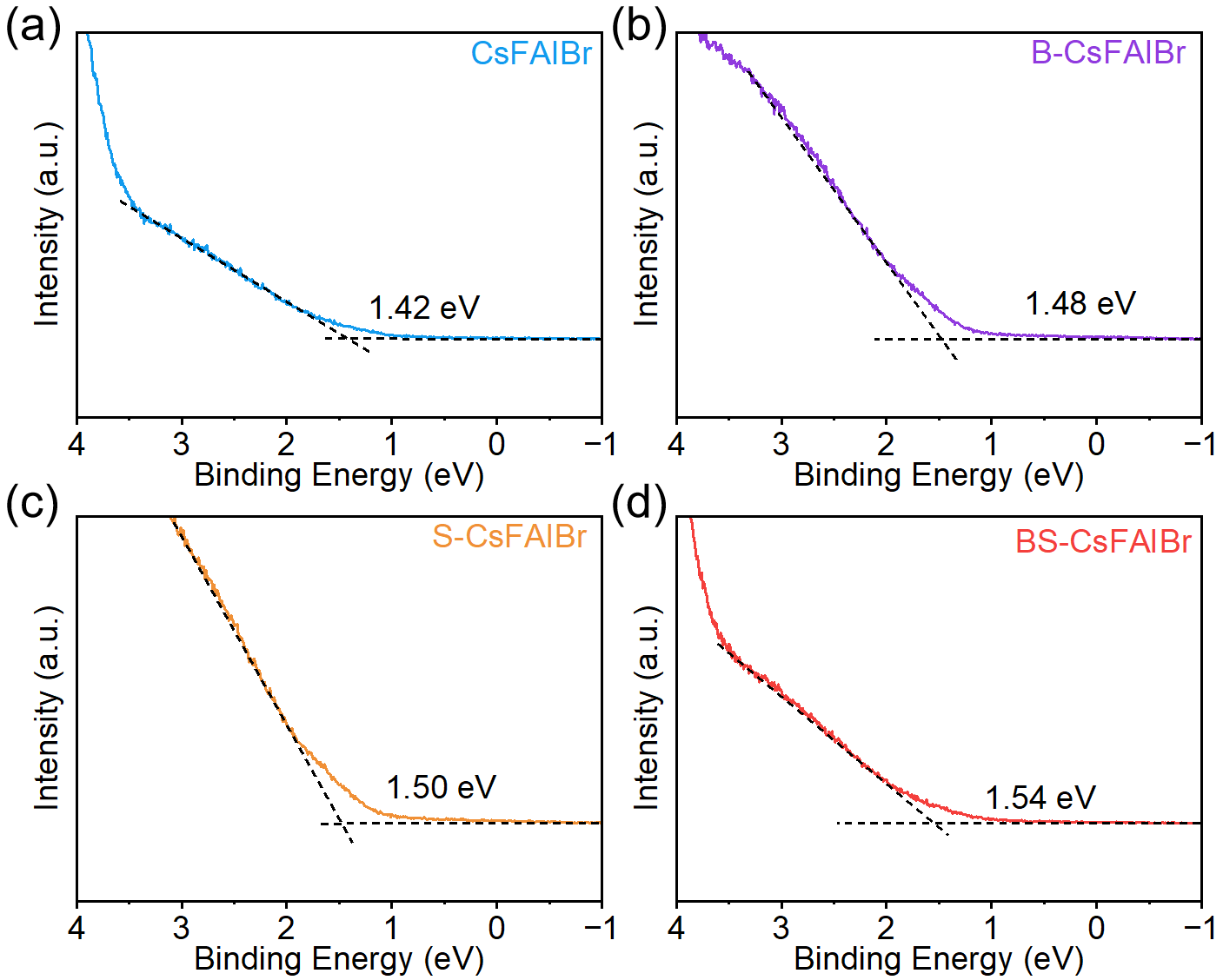
**

**Figure S26.** The secondary electron cutoff for CsFAIBr, B-CsFAIBr, S-CsFAIBr, and BS-CsFAIBr perovskite films.

**
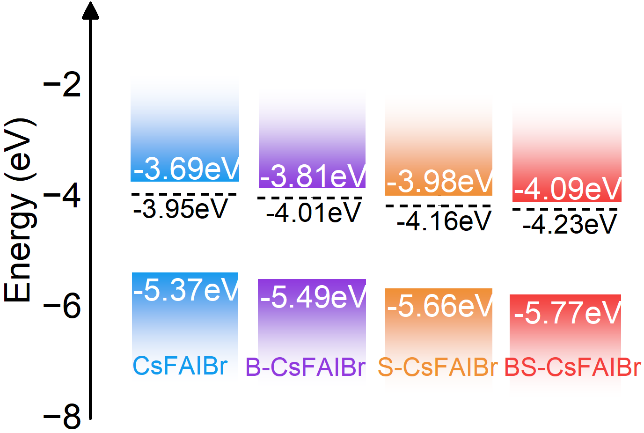
**

**Figure S27.** Schematic diagram of the energy level of perovskite films at the time of CsFAIBr, B-CsFAIBr, S-CsFAIBr, and BS-CsFAIBr.


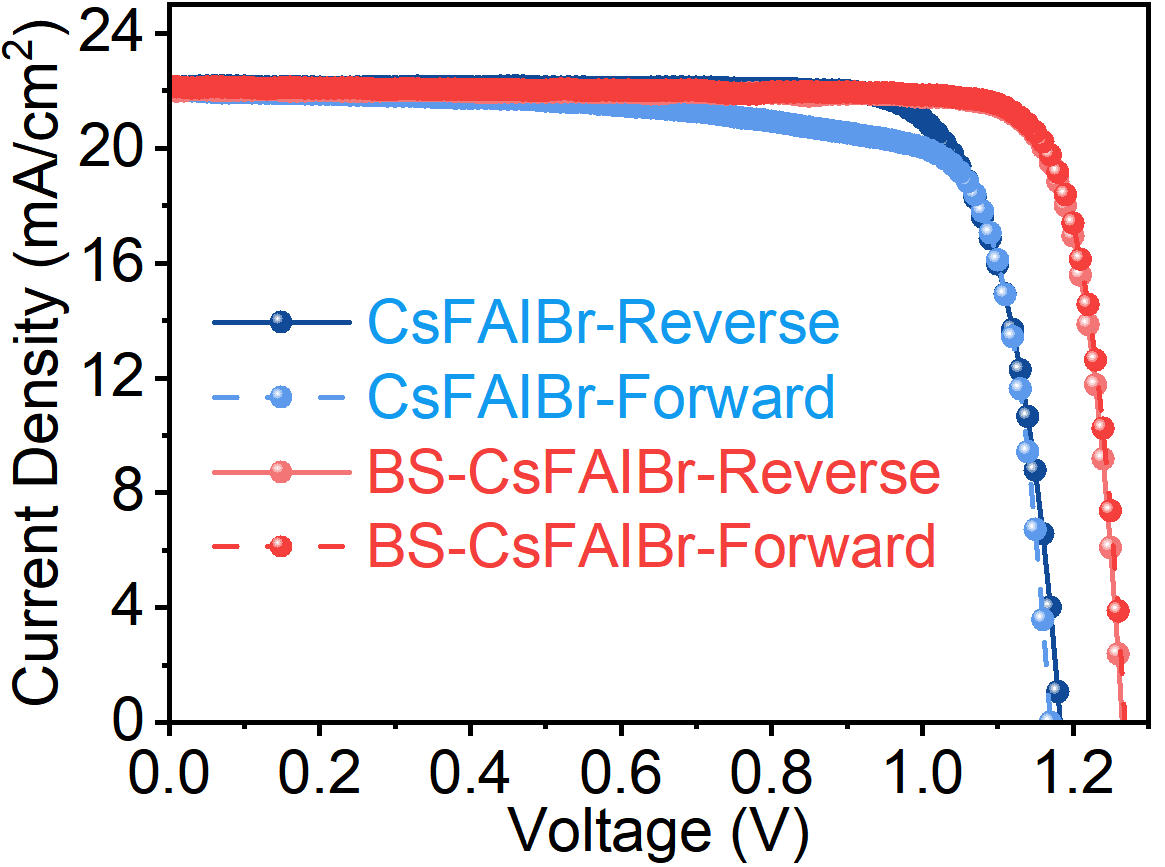


**Figure S28.** Typical *J*-*V* curves for CsFAIBr- and BS-CsFAIBr- based devices.

**
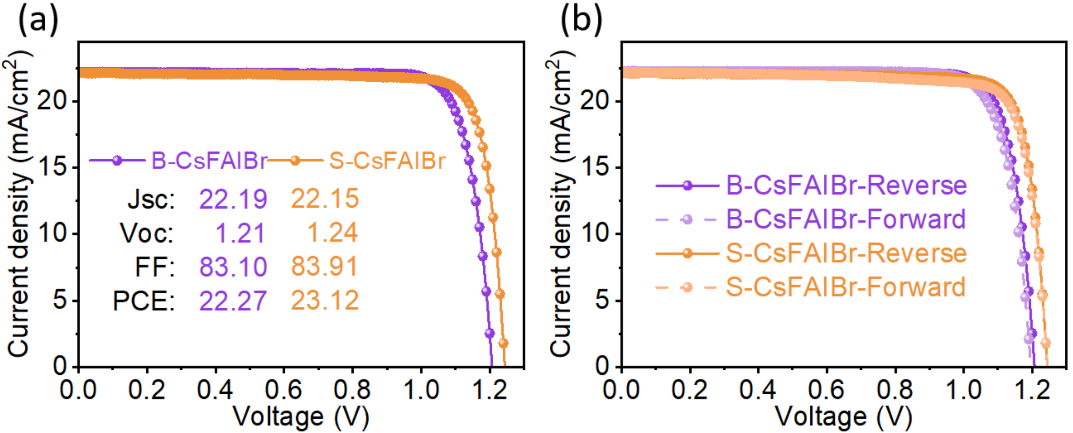
**

**Figure S29.** Typical J-V curves for B-CsFAIBr and S-CsFAIBr based devices and the forward and reverse hysteresis curves.


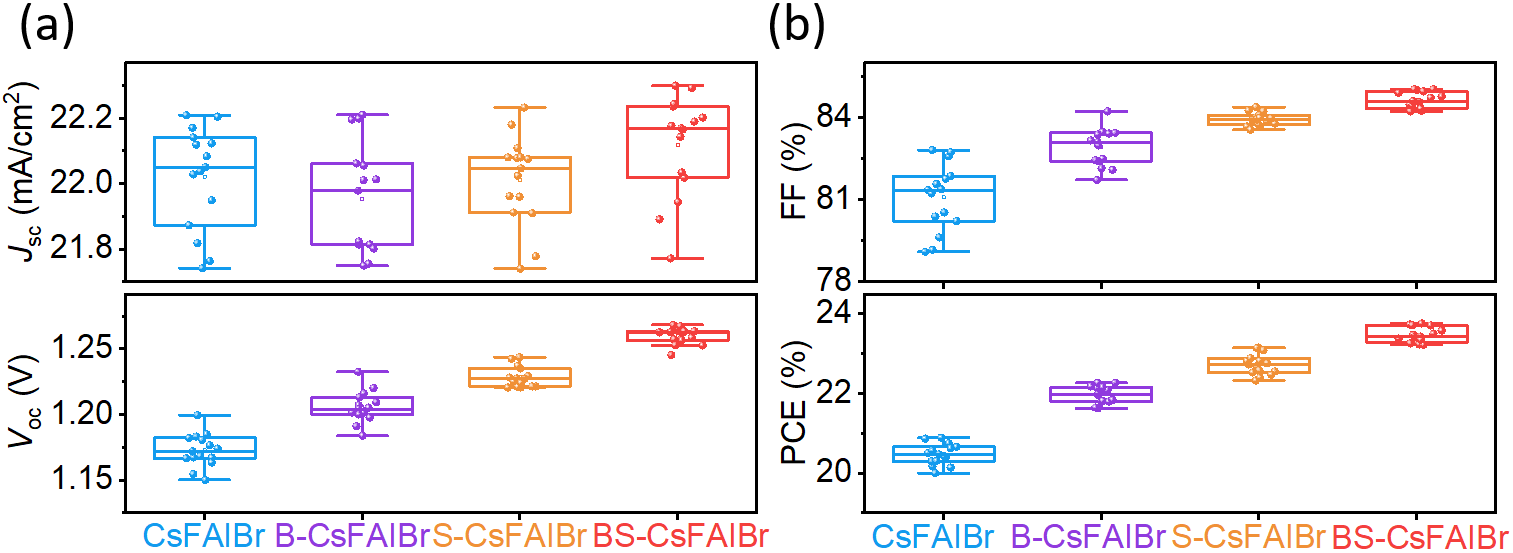


**Figure S30**. Statistical distribution of a) *J*_sc_ and *V*_oc_ and b) PCE and FF for CsFAIBr, B-CsFAIBr, S-CsFAIBr, and BS-CsFAIBr based devices.

**
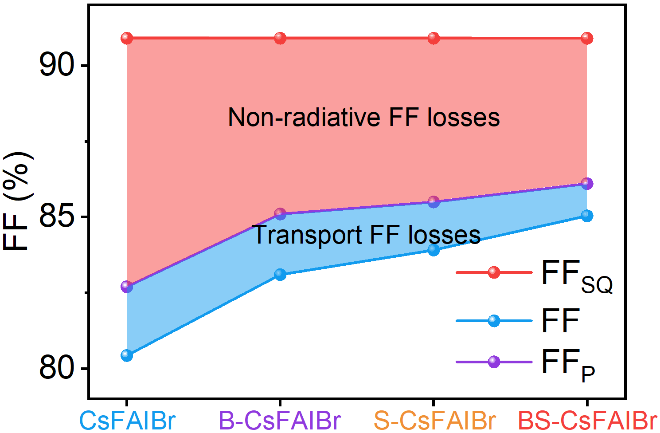
**

**Figure S31.** The comparison diagram of S-Q limit *FF*, pseudo-*FF*, and actual *FF*.

**
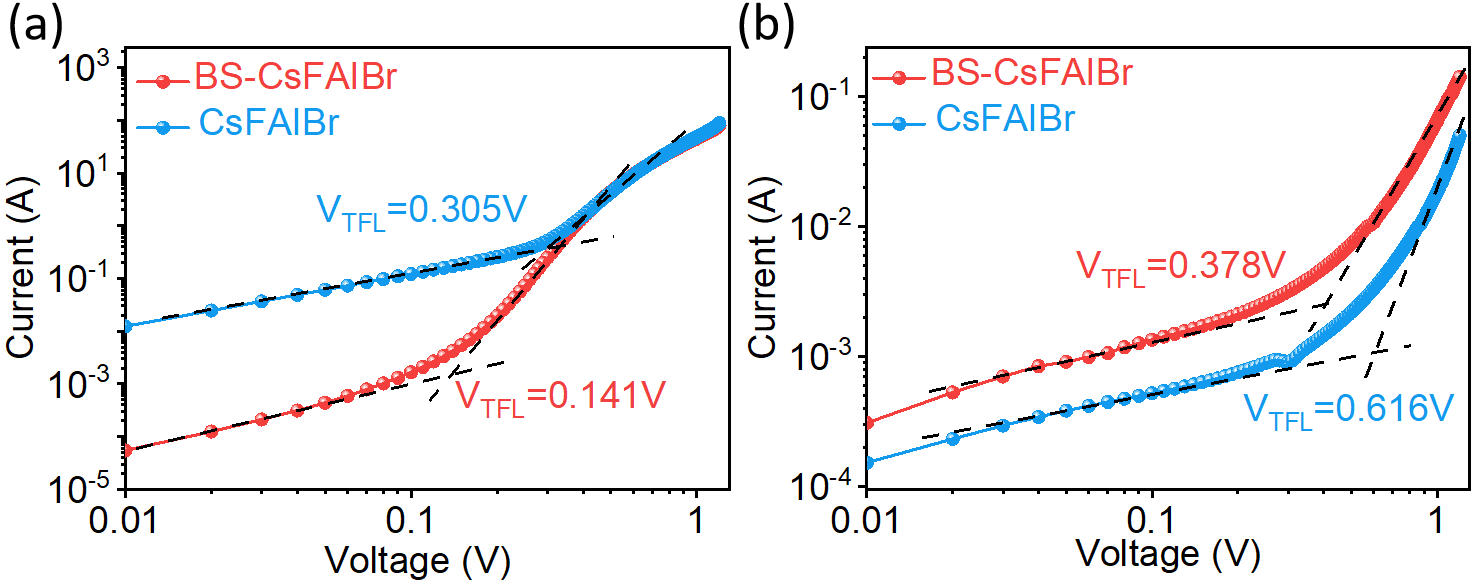
**

**Figure S32.** a) SCLC curves of the electronic-only devices (ITO/SnO_2_/perovskite/C_60_/BCP/Ag). b) hole-only devices (ITO/4PACDB/perovskite/Spiro/Ag).

**
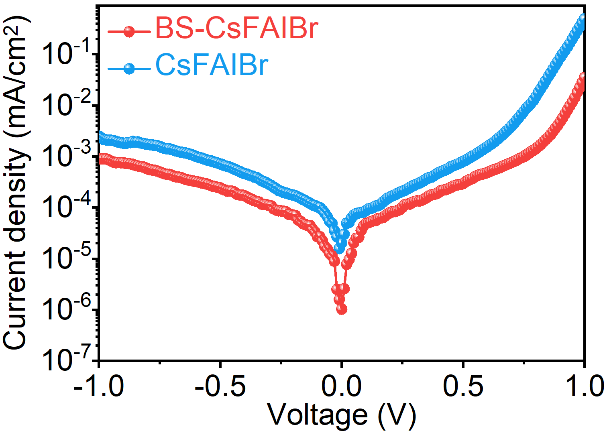
**

**Figure S33.** Dark *J*–*V* curves of CsFAIBr and BS-CsFAIBr.


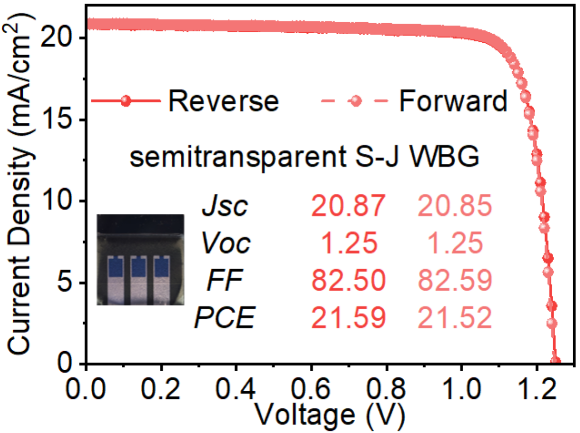


**Figure S34.** *J-V* curve of a semi-transparent single-junction (S-J) WBG.
